# Kirigami‐Structured, Low‐Impedance, and Skin‐Conformal Electronics for Long‐Term Biopotential Monitoring and Human–Machine Interfaces

**DOI:** 10.1002/advs.202304871

**Published:** 2023-11-20

**Authors:** Meili Xia, Jianwen Liu, Beom Jin Kim, Yongju Gao, Yunlong Zhou, Yongjing Zhang, Duxia Cao, Songfang Zhao, Yang Li, Jong‐Hyun Ahn

**Affiliations:** ^1^ School of Materials Science and Engineering University of Jinan Jinan 250022 China; ^2^ School of Information Science and Engineering University of Jinan Jinan 250022 China; ^3^ School of Electrical and Electronic Engineering Yonsei University Seoul 03722 Republic of Korea; ^4^ Shandong Zhongke Advanced Technology Co., Ltd Jinan 250000 China; ^5^ School of Microelectronics Shandong University Jinan 250101 China

**Keywords:** bioelectric interfacial impedance, epidermal electrode, epidermal electrophysiology, human–machine interface, Kirigami structure

## Abstract

Epidermal dry electrodes with high skin‐compliant stretchability, low bioelectric interfacial impedance, and long‐term reliability are crucial for biopotential signal recording and human–machine interaction. However, incorporating these essential characteristics into dry electrodes remains a challenge. Here, a skin‐conformal dry electrode is developed by encapsulating kirigami‐structured poly(3,4‐ethylenedioxythiophene):poly(styrene sulfonate) (PEDOT:PSS)/polyvinyl alcohol (PVA)/silver nanowires (Ag NWs) film with ultrathin polyurethane (PU) tape. This Kirigami‐structured PEDOT:PSS/PVA/Ag NWs/PU epidermal electrode exhibits a low sheet resistance (≈3.9 Ω sq^−1^), large skin‐compliant stretchability (>100%), low interfacial impedance (≈27.41 kΩ at 100 Hz and ≈59.76 kΩ at 10 Hz), and sufficient mechanoelectrical stability. This enhanced performance is attributed to the synergistic effects of ionic/electronic current from PEDOT:PSS/Ag NWs dual conductive network, Kirigami structure, and unique encapsulation. Compared with the existing dry electrodes or standard gel electrodes, the as‐prepared electrodes possess lower interfacial impedance and noise in various conditions (e.g., sweat, wet, and movement), indicating superior water/motion‐interference resistance. Moreover, they can acquire high‐quality biopotential signals even after water rinsing and ultrasonic cleaning. These outstanding advantages enable the Kirigami‐structured PEDOT:PSS/PVA/Ag NWs/PU electrodes to effectively monitor human motions in real‐time and record epidermal biopotential signals, such as electrocardiogram, electromyogram, and electrooculogram under various conditions, and control external electronics, thereby facilitating human–machine interactions.

## Graphical abstract

A skin‐conformal dry electrode with low sheet resistance, large stretchability, low interfacial impedance, and sufficient mechanoelectrical stability, is developed by encapsulating Kirigami‐structured poly(3,4‐ethylenedioxythiophene):poly(styrene sulfonate)/polyvinyl alcohol/silver nanowires film with ultrathin polyurethane tape. This achievement has facilitated important applications in monitoring human motions, record epidermal biopotential signals with superior water/motion‐interference resistance, and control external electronics, thereby facilitating human–machine interactions.

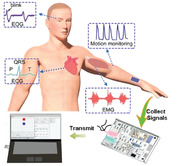

## 1 Introduction

The rapid advancement of artificial intelligence (AI) has drawn significant attention to epidermal electronics, given their promising applications in clinical diagnostics/therapeutics, athletic training, and human–machine interfaces (HMI).^[^ ^1^, ^2^, ^3^, ^4^, ^5^ ^]^ These applications require a seamless integration of epidermal electronics with human skin. Especially, electrophysiological signals, such as electromyogram (EMG), electrocardiogram (ECG), electroencephalogram (EEG), and electrooculogram (EOG), predominantly rely on ionic conduction. The universal nature and unique characteristics of these signals make them highly valuable for active electrophysiological interactions in HMI.^[^ ^6^, ^7^, ^8^ ^]^ Therefore, it is crucial to effectively collect and transmit these ionic signals with long‐term reliability, requiring epidermal electrodes to effectively interface with ionic fluxes in electrolytic tissues and electronic current in external devices. Establishing seamless and compliant interfaces between epidermal electronics and target tissues become paramount to ensure the reliable function of HMI through electrophysiological mode.^[^ ^9^ ^]^

Ion‐conducting gel electrodes, such as hydrogels and organogels, have been extensively used in bioelectronics and HMI due to their ability to transmit and collect high‐quality bioelectric signals through ionic conduction.^[^ ^4^, ^9^, ^10^, ^11^ ^]^ For example, Pan et al. reported a compliant ionic adhesive electrode based on alginate‐polyacrylamide hydrogels for recording dynamically weak surface EMG signals with minimal crosstalk.^[^ ^7^ ^]^ Luo et al. demonstrated a self‐adaptive HMI based on MXene cross‐linked ion‐conducting poly (sodium acrylate) hydrogel for active EEG interaction.^[^ ^9^ ^]^ Wang et al. reported an on‐skin paintable conductive organogel for long‐term high‐quality EEG recording.^[^ ^12^ ^]^ However, the dehydration of hydrogels can create gaps that degrade the coupling of ionic and electronic currents, resulting in high interfacial impedance, which leads to noise or false signals. In addition, the swelling and low adhesion of the hydrogels in humidity can cause electrode displacement and significant motion artifacts, leading to poor electrophysiological signal resolution. Furthermore, conductive ions in gelled electrodes can be exchanged or leaked in humid environments, further increasing the interfacial impedance.^[^ ^13^, ^14^ ^]^ To overcome these limitations, dry electrodes that offer excellent compliance with deformed skin, sufficiently mechanoelectrical stability, and good biocompatibility are highly desired for long‐term biopotential monitoring and HMI.

Recently, various conductive materials, including conductive polymers, gold film, and liquid metal, have been introduced into epidermal dry electrodes for electrophysiological signal recording.^[^ ^13^, ^15^, ^16^ ^]^ For example, Tang et al. reported a sticky electrode composed of gallium indium alloy and acrylate polymer adhesives for surface EMG recording.^[^ ^8^ ^]^ However, metal‐based electrodes suffer from high interfacial impedance due to the mismatch between ionic and electronic currents during the coupling process and their low charge density.^[^ ^17^ ^]^ As a promising alternative, poly (3,4‐ethyllenedioxythiophene): poly (styrene sulfonate) (PEDOT:PSS) is a commercially available conductive polymer with tunable rheological behavior, tunable electrical performance, and bio‐compatibility.^[^ ^4^, ^18^, ^19^ ^]^ Chemical vapor deposition‐grown graphene covered with PEDOT:PSS layer has been utilized to create an ultra‐conformal dry electrode for long‐term electrophysiological signal recording with minimal motion artifacts.^[^ ^20^ ^]^ Nevertheless, the limited stretchability of PEDOT:PSS restricts its applications on largely deformed skin, and the interfacial impedance (≈32 kΩ at 100 Hz and ≈80 kΩ at 10 Hz) still exceeds the ideal impedance range for the electrode–skin system (6–10 kΩ). Doping rigid PEDOT:PSS composites with a biocompatible supramolecular solvent has been explored to create a self‐adaptive conductive polymer composite with low modulus and high stretchability. However, this approach often results in poor conductivity (1–37 S cm^−1^), and the effective contact area between the conductive materials and skin is limited, leading to suboptimal EMG signal quality.^[^ ^4^ ^]^ Despite some promising bioelectric interfaces achieved through recent breakthroughs in microfabrication and bioelectronics, significant efforts are still required to develop dry electrodes with large stretchability, excellent mechanoelectrical stability, low interfacial impedance, long‐term reliability, and good biocompatibility.

Kirigami structures with a pattern of cuts enable large stretchability through mesoscale bending and twisting of each beam. They also offer the capability of large‐area integration on three‐dimensional dynamic surfaces.^[^ ^15^, ^21^, ^22^ ^]^ However, the cuts in the Kirigami structure can lead to stress concentration during deformation, which should be considered. Herein, we present a highly stretchable, low interfacial impedance, and skin‐conformal epidermal electrode with a Kirigami structure for long‐term biopotential monitoring and HMI. Silver nanowires (Ag NWs) were deposited on a PEDOT:PSS/polyvinyl alcohol (PVA) film using a spray‐coating approach, and the Kirigami‐structured epidermal electrodes were fabricated through Kirigami cutting, followed by transfer onto ultrathin medical polyurethane (PU) tape. The optimized dry epidermal electrodes, named as Kirigami‐structured PEDOT:PSS/PVA/Ag NWs/PU (KPPAP) electrodes, exhibited a low sheet resistance of ≈3.9 Ω sq^−1^, large skin‐compliant stretchability of >100%, and a low interfacial impedance of ≈27.41 kΩ at 100 Hz and ≈59.76 kΩ at 10 Hz. These electrodes demonstrated sufficient mechanoelectrical stability, attributed to the synergistic effects of capacitive coupling process between ionic and electronic currents facilitated by Ag NWs and PEDOT:PSS, Kirigami structure, and unique encapsulation. These Kirigami‐structured PEDOT:PSS/PVA/Ag NWs/PU electrodes exhibited outstanding capacity for real‐time human motion monitoring, and recording of various epidermal biopotential signals, including ECG, EMG, and EOG, exhibiting robust and excellent signal quality. Moreover, the collected EMG and EOG signals were successfully used to control external electronics, thereby achieving human–machine interactions. This study demonstrates the superiority of advanced materials and structures in the development of high‐performance epidermal electrodes.

## 2 Results and Discussion

### 2.1 Design and Fabrication of Kirigami‐Structured PEDOT:PSS/PVA/Ag NWs/PU Skin‐Conformal Epidermal Electrodes

Electrophysiology plays an important role in future wisdom medicine and AI device control, which requires the skin‐conformal epidermal electrodes to possess low bioelectric interfacial impedance, realizing the capacitive coupling process of ionic and electronic current. Moreover, the epidermal electrodes should be stretchable to achieve conformable attachment to human skin during deformation, reducing the motion artifacts. Hence, an ideal epidermal electrode with large stretchability, excellent mechanoelectrical stability, low bioelectric interfacial impedance, and long‐term reliability is capable of addressing this issue.^[^ ^19^, ^23^, ^24^ ^]^

**Figure 1:**
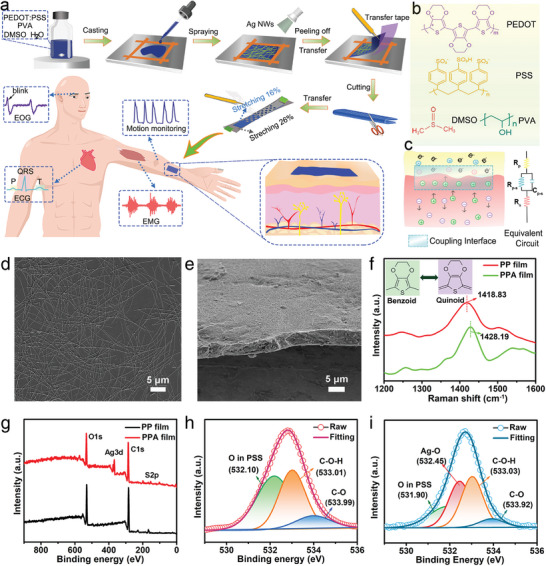
Facile fabrication of Kirigami‐structured PEDOT:PSS/PVA/Ag NWs/PU electrodes and structural characterizations of PEDOT:PSS/PVA/Ag NWs films. a) Schematic fabrication process of the Kirigami‐structured PEDOT:PSS/PVA/Ag NWs/PU electrodes and the Kirigami‐structured PEDOT:PSS/PVA/Ag NWs/PU electrodes on the skin for epidermal biopotential signal recording and human motion monitoring. b) Chemical structure of PEDOT:PSS, DMSO, and PVA. c) Schematic of the coupling process of the ionic fluxes in electrolytic tissues and electronic current in the recording electrode, and the equivalent circuit model of the skin–electrode interface (*R*_P_ is the resistance of electrode, *R*_P‐S_ is the resistance of interface, *C*_P‐S_ is the capacitance of the interface, *R*_S_ is the skin resistance). d) Scanning electron microscopy (SEM) image of Ag NWs. e) Cross‐sectional SEM image of the PEDOT:PSS/PVA/Ag NWs (PPA) film. f) Raman spectra of the PEDOT:PSS/PVA (PP) film and PEDOT:PSS/PVA/Ag NWs film. g) XPS survey spectra of PEDOT:PSS/PVA film and PEDOT:PSS/PVA/Ag NWs film (Ag NWs side). h,i) XPS spectra of O1s in the PEDOT:PSS/PVA film (h) and PEDOT:PSS/PVA/Ag NWs film (i).

**Figure** **1**a shows the detailed fabrication process of Kirigami‐structured PEDOT:PSS/PVA/Ag NWs/PU epidermal electrodes. PEDOT:PSS was selected because of its fast electrochemical kinetics, high conductivity, and biocompatibility. Especially, the high electron/ion mobility and rapid electrochemical dynamics of PEDOT: PSS electrodes can enhance the coupling process of electrons and ions at the device/skin interface. To further enhance the conductivity of PEDOT:PSS, the secondary dopant such as ethylene glycol (EG) or dimethyl sulfoxide (DMSO) are generally introduced.^[^ ^25^, ^26^ ^]^ In this study, DMSO was added as a secondary dopant to PEDOT:PSS solution, which can prompt the benzene‐quinone transition due to the interactions between DMSO and PSS.^[^ ^27^ ^]^ Moreover, the incorporation of PVA into PEDOT:PSS film improves its mechanical properties (Figure 1b). To realize the highly efficient coupling process of ionic current in tissue and electronic current in recording devices, a layered configuration is designed by depositing Ag NWs with a length of 15–35 µm and diameter of 30–50 nm on PEDOT:PSS/PVA film (PP film), and the thickness of the PEDOT:PSS/PVA/Ag NWs film (PPA film) is ≈4.10 µm (Figure 1d,e; Figure S1, Supporting Information). The PEDOT:PSS can transfer electrons and ions owing to its large conjugate structure and ion pairs, whereas Ag NWs can transfer electrons. Therefore, the rational design of PEDOT:PSS and Ag NWs in PEDOT:PSS/PVA film can provide dual transfer pathways for ion and electron transport, leading to more efficient conduction and acquisition of bioelectric signals. A Kirigami structure was incorporated into the PEDOT:PSS/PVA/Ag NWs film, which was then transferred onto the PU tape. It is worth noting that PEDOT:PSS has the ability to transmit bioelectric signals through mixed ions/electrons, similar to the ionic conduction in the biological tissue.^[^ ^28^ ^]^ Therefore, the PEDOT:PSS/PVA side on the Kirigami‐structured PEDOT:PSS/PVA/Ag NWs/PU electrodes should be contacted with the human skin.^[^ ^29^, ^30^ ^]^ The equivalent circuit model of the skin–electrode interface (Figure 1c) demonstrates the coupling process of the ionic fluxes in electrolytic tissue media and electronic current in the recording electrode,^[^ ^7^ ^]^ which is confirmed by the excellent fit of the simulated values with the experimental values in Nyquist diagram and the interfacial impedance (Figure S2, Supporting Information). Because of the sufficient adhesion and stretchability of the Kirigami‐structured PEDOT:PSS/PVA/Ag NWs/PU electrodes, they can conform well with the human skin under diverse deformation, and are utilized as stretchable epidermal electrodes for monitoring physiological signals, including human motions and epidermal biopotentials.

To confirm the interaction between PEDOT:PSS/PVA film and Ag NWs, Raman spectroscopy and X‐ray photoelectron spectroscopy (XPS) were used to conduct a comprehensive study on the PEDOT:PSS/PVA/Ag NWs film. There is a vibration peak at 1418.83 cm^−1^ associated with the symmetric stretching of the conjugated C═C in the cyclic thiophene ring of PEDOT chain, and the corresponding peak position is blueshifted to 1428.19 cm^−1^ in PEDOT:PSS/PVA/Ag NWs film after the introduction of Ag NWs (Figure 1f). The blueshift indicates the strong interactions between PEDOT:PSS/PVA and Ag NWs, which facilitates the effective transport of ions and electrons during electrochemical reaction by the delocalization of *π* electrons. Moreover, the strong interaction endows the thiophene rings on PEDOT chains with transition from a coil‐like benzoid state to a linear‐like quinoid state, and facilitates the inter‐ and intra‐chain charge transport, which enables electrons/ions to hop from the conductive PEDOT chains to the Ag NWs or from Ag NWs to the PEDOT chains.^[^ ^31^ ^]^ The bonding states and chemical compositions of the PEDOT:PSS/PVA/Ag NWs film associated with the structural superiority were further investigated by XPS. As shown in Figure 1g, C, O, S, and Ag elements exist in the PEDOT:PSS/PVA/Ag NWs film. The high‐resolution O1s spectra of the PEDOT:PSS/PVA film are deconvoluted into three peaks located at 532.10, 533.01, and 533.99 eV, which are attributed to O atoms in the sulfonic acid group, C–O–H group in PVA, and C–O group in PEDOT, respectively (Figure 1h).^[^ ^32^ ^]^ In contrast, four peaks located at 531.90, 532.45, 533.03, and 533.92 eV, respectively, are deconvoluted in O1s spectra of the PEDOT:PSS/PVA/Ag NWs film (Figure 1i). Compared with those peaks in the PEDOT:PSS/PVA film, a new peak appears at a binding energy of 532.45 eV, which is resulted from the interaction between silver and oxygen atoms. This observation is consistent with the result obtained from the analysis of Raman spectra. Because of the higher binding energy of the new peak (Ag–O, 532.45 eV) than that of the oxygen atom in the sulfonic acid group (531.90 eV), the sulfonate moiety in the PSS can afford electrons to the adjacent silver atoms, and thus coordinates with the Ag NWs.^[^ ^33^ ^]^ The high‐resolution C1s spectra are decomposed into three peaks at 284.80, 286.39, and 288.41 eV, which correspond to C–C/C═C, C–O–C/C‐O‐H, and C–S bonds, respectively. No obvious changes in C1s spectra of PEDOT:PSS/PVA and PEDOT:PSS/PVA/Ag NWs films are observed (Figure S3, Supporting Information).^[^ ^34^ ^]^ Because of different chemical environments, two different sulfur functional groups are observed in the S2p spectra. The four deconvoluted peaks in PEDOT:PSS/PVA film at 163.81, 165.01, 168.31, and 169.51 eV are attributed to S–C 2p_3/2_ and S–C 2p_1/2_ in PEDOT_,_ and S–O 2p_3/2_ and S–O 2p_1/2_ in PSS, respectively.^[^ ^35^ ^]^ However, these deconvoluted peaks in PEDOT:PSS/PVA/Ag NWs film shift to the lower binding energies (162.15, 163.35, 167.95, and 168.15 eV), respectively. These above‐mentioned peak shifts indicate the interfacial interaction between PEDOT:PSS or PVA and Ag NWs. Owing to the layered structure of PEDOT:PSS/PVA/Ag NWs film and low sample depth of XPS (1–6 nm), the XPS spectra of the PEDOT:PSS/PVA side on the PEDOT:PSS/PVA/Ag NWs film is also investigated (Figure S4, Supporting Information). The peak of C1s in S‐C shifts to 287.31 from 288.41 eV, and the peak area ratio of C1s between PSS and PEDOT decreases to 1.56 from 1.81. These obvious changes also indicate the formation of the interaction between Ag NWs and PSS, and the reduction of the interaction between PEDOT and PSS, thereby enhancing the benzene‐quinone transition and conductivity of PEDOT:PSS.

The characterizations mentioned above confirm the presence of a strong interfacial interaction between the PEDOT:PSS/PVA film and Ag NWs. This interaction prevents the detachment of Ag NWs during applications and enhances the ion/electron transport between the components. As a result, there is a higher charge density at the bioelectronic interface, and the interfacial impedance with the skin is reduced.

### 2.2 Electrical, Electrochemical Performance, and Biocompatibility of the PEDOT:PSS/PVA/Ag NWs Films

**Figure 2:**
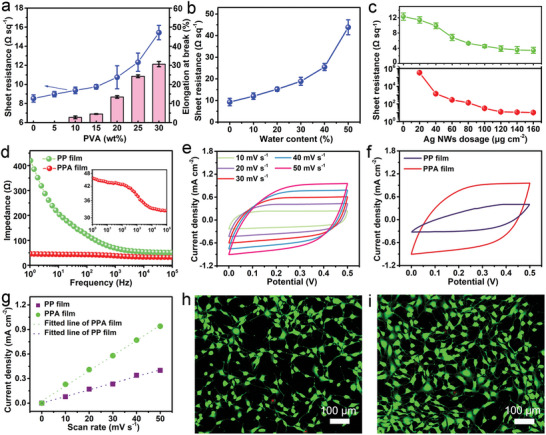
Electrical and electrochemical properties and biocompatibility. a) Sheet resistance and elongation at break of PEDOT:PSS/PVA (PP) films with different PVA loadings. b) Sheet resistance of Ag NWs films when spraying Ag NWs/ethanol solution with different water contents. c) Sheet resistance of Ag NWs films (bottom) and PEDOT:PSS/PVA/Ag NWs (PPA) films (top) with different dosages of Ag NWs. d) Electrochemical impedance spectroscopy (EIS) of PEDOT:PSS/PVA films and PEDOT:PSS/PVA/Ag NWs films with a 1 cm×1 cm area as electrodes in 0.01 m phosphate buffer saline (PBS). e) Cyclic voltammograms (CVs) of PEDOT:PSS/PVA/Ag NWs films as electrodes at different scan rates in 0.01 m PBS. f) CVs of PEDOT:PSS/PVA films and PEDOT:PSS/PVA/Ag NWs films as electrodes at a scan rate of 50 mV s^−1^. g) Linear relationship between charging current densities and scan rates for PEDOT:PSS/PVA films and PEDOT:PSS/PVA/Ag NWs films as electrodes in 0.01 m PBS. h,i) Fluorescent images of stained NIH 3T3 cells cultured on control film (h) and PEDOT:PSS/PVA/Ag NWs film (i) after 24 h. Live cells: green. Dead cells: red.

Because of the limited intrinsic stretchability of as‐cast PEDOT:PSS films, which typically exhibit only ≈2% elongation at break, the optimization of the conductivity and mechanical properties of PEDOT:PSS film becomes an important and challenging task. This is primarily due to the trade‐off between mechanical flexibility and conductivity.^[^ ^36^ ^]^ Here, doping modification was implemented by blending the PEDOT:PSS solution with plasticizer or high‐loading soft polymers. This approach aimed to enhance the mechanical properties, specially the elongation at break, and conductivity of the as‐cast PEDOT:PSS films. A concentration of 3 wt.% DMSO was generally selected to enhance cohesion and electrical conductivity of PEDOT:PSS.^[^ ^27^ ^]^ Although high conductivity is obtained by the addition of DMSO, the mechanical flexibility of the as‐cast PEDOT:PSS films is still insufficient for meeting the subsequent requirements of tailoring process (Figure S5a, Supporting Information). To address this limitation, PVA was chosen to improve the mechanical properties owing to its good miscibility, excellent mechanical properties and biocompatibility. Stress‐strain curves of the PEDOT:PSS/PVA films with different loadings of PVA are shown in Figure S5b (Supporting Information). As the PVA loading increases, the elongation at break also increases. For example, the PEDOT:PSS/PVA film with a 10 wt.% of PVA exhibits rigidity with 2.90% of elongation at break and 35.52 MPa of fracture stress, while the PEDOT:PSS/PVA film with a 30 wt.% of PVA shows softness with 37.02% of elongation at break and 19.72 MPa of fracture stress (**Figure** **2**a). Therefore, the mechanical flexibility of PEDOT:PSS/PVA films can be adjusted by modifying the PVA content.

In addition to acting as a soft material, the presence of PVA also affects the conductivity of the PEDOT:PSS/PVA film. The conductivity of the film follows percolation theory, which is attributed to the electrical insulative properties of PVA. The sheet resistance of the PEDOT:PSS/PVA films with different loadings of PVA was investigated (Figure 2a). The conductivity exhibits a smooth initial decrease, followed by a sharp decrease beyond a PVA loading of 20 wt.%. To achieve a balanced performance in both mechanical flexibility and conductivity, PEDOT:PSS/PVA films with a 25 wt.% PVA loading were chosen for subsequent experiments. To enhance conductivity, Ag NWs were spray‐coated onto the PEDOT:PSS/PVA film.

Introducing a strong airflow while spraying serves to expedite solvent evaporation and the corresponding high velocity impact of the liquid drop can generate mechanical loading to endow adjacent Ag NWs with compacted junctions (Figure S1, Supporting Information), resulting in high conductivity.^[^ ^37^ ^]^ Surface tension of Ag NWs ink was adjusted by tuning the volume ratio of water (72.75 mN m^−1^)/ethanol (22.32 mN m^−1^) in Ag NWs/ethanol suspension. The sheet resistance of the pure Ag NWs films increases with the increase of water contents owing to the formation of a conventional random Ag NWs network (Figure 2b). Moreover, the addition of water to the Ag NWs ink enhances its surface tension and boiling point. This unique property allows the droplets to evaporate slowly and facilitates free movement, enabling the Ag NWs to distribute randomly on the substrate. As the water content increases, the density of conventional random network increases (Figure S6, Supporting Information). As a result, the conductivity is reduced. Additionally, the introduction of water effectively enhances the interfacial interaction between the Ag NWs and PEDOT:PSS/PVA films. This was evaluated through a peeling test using 3M adhesive tape (Figure S7, Supporting Information). In the absence of water in Ag NWs ink, Ag NWs are transferred onto the tape and easily detached from the PEDOT:PSS/PVA film. The introduction of water can facilitate the embedding of Ag NWs into the PEDOT:PSS/PVA film owing to the absorption of water by PEDOT:PSS/PVA film, therefore, unobvious interface between Ag NWs and PEDOT:PSS/PVA film is generated, improving interfacial interaction. High electrical conductivity can be achieved by increasing the density of Ag NWs (Figure 2c). When the density of Ag NWs is 120 µg cm^−2^, the sheet resistance of pure Ag NWs reaches 12.85 Ω sq^−1^, and then changes slightly as the density of Ag NWs increases. Similar trend is observed in PEDOT:PSS/PVA/Ag NWs film. Therefore, the PEDOT:PSS/PVA/Ag NWs films with optimized performance are fabricated at the following conditions: 3 wt.% of DMSO doping PEDOT:PSS, 25 wt.% of PVA blending with PEDOT:PSS, 20 Vol.% of water in Ag NWs ink, and 120 µg cm^−2^ of Ag NWs dosage. Compared with the sheet resistance (12.33 Ω sq^−1^) of the PEDOT:PSS/PVA film, the PEDOT:PSS/PVA/Ag NWs film possesses a lower sheet resistance (3.90 Ω sq^−1^), indicating excellent conductivity (Figure S8, Supporting Information). Moreover, the number of stacking layers of Ag NWs is ≈2–4, with six layers of stacking being rare, the diameter of Ag NWs is 30–50 nm (Figure S9, Supporting Information). Therefore, the maximum stacking thickness of Ag NWs on PEDOT:PSS/PVA film is ≈300 nm, which has a little effect on the results. According to the formula: *σ* = 1/(*R*_sh_×*t*), the conductivity of PEDOT:PSS/PVA/Ag NWs and PEDOT:PSS/PVA films is 625.39 and 207.95 S cm^−1^, indicating the improvement by the introduction of Ag NWs. This is because PEDOT:PSS and Ag NWs can provide more conductive pathways to transfer electrons/ions, and the PEDOT:PSS/PVA conductive substrate as bridge can avoid the incomplete connections between Ag NWs, resulting in high conductivity.^[^ ^38^ ^]^

Epidermal electrodes perform ion‐to‐electron signal transduction and transmit the biopotential signals to the recording devices, and the capacitive coupling process of ionic and electronic current at the tissue‐electrode interface is vital for the quality of the collected biopotential signals. Therefore, their electrochemical properties including electrochemical impedance, charge storage capacity (CSC), and specific capacitance, were investigated for collecting or delivering biopotential signals.^[^ ^39^, ^40^ ^]^ Compared with the impedance of PEDOT:PSS/PVA film, the PEDOT:PSS/PVA/Ag NWs film exhibits lower impedance over the entire frequency range (1–10^5^ Hz), and their difference becomes larger in the low‐frequency region (1–10^3^ Hz) (Figure 2d). In addition, the plot of impedance phase angle versus frequency indicates that the PEDOT:PSS/PVA/Ag NWs film behaves more like a resistor (Figure S10, Supporting Information).^[^ ^41^ ^]^ The Bode plots clearly indicate the PEDOT:PSS/PVA/Ag NWs films have low impedance and high conductivity for bioelectrical signal recording. To confirm the better performance in charge transfer, cyclic voltammetry (CV) was conducted at different scan rates (Figure 2e,f; Figure S10, Supporting Information). The CV curve of PEDOT:PSS/PVA/Ag NWs film still remains rectangular in shape at a fast scan rate of 50 mV s^−1^, confirming the formation of an efficient electric double layer (EDL) and fast charge transportation within the PEDOT:PSS/PVA/Ag NWs films.^[^ ^42^ ^]^ In contrast, the CV curves of PEDOT:PSS/PVA film lose rectangular shape at a scan rate of 30 mV s^−1^. Moreover, the PEDOT:PSS/PVA/Ag NWs film has a charge storage capacity per unit area of 11.98 mC cm^−2^ at a scan rate of 50 mV s^−1^, which is much larger than 3.98 mC cm^−2^ of PEDOT:PSS/PVA film. Large CSC values indicate that PEDOT:PSS/PVA/Ag NWs films can mediate the transfer of more electrical and ionic charge carriers. Such excellent charge transfer ability can decrease the dimensions of the electrodes, thus minimizing the crosstalk from irrelevant muscles.^[^ ^43^ ^]^ There is a linear relationship between the charging current densities and scan rates in the range of 10–50 mV s^−1^ (Figure 2g), also demonstrating the formation of EDL capacitance at the electrode/electrolyte interface. The specific capacitance of the PEDOT:PSS/PVA/Ag NWs film is calculated to be 18.54 mF cm^−2^, which is larger than that of the PEDOT:PSS/PVA film (8.14 mF cm^−2^). Similar results are observed at a scanning potential range of 0–0.8 V (Figure S10, Supporting Information). These improved electrochemical performance confirms the superiority of the layer‐structured PEDOT:PSS/PVA/Ag NWs film in reducing the impedance and improving the charge storage capacity, which is strongly demanded for epidermal electrodes to record or deliver biopotential signals.^[^ ^17^ ^]^ Furthermore, ensuring the safety and biocompatibility of epidermal electrodes is crucial for intimate contact with the skin. This was confirmed through a live/dead assay conducted on NIH 3T3 fibroblasts cultured on a PEDOT:PSS/PVA/Ag NWs film, along with a blank control group (Figure 2h,i). After 24 h of cell culturing, the cell viability reached 99%, which is comparable to that of the blank control group. Even after 72 h, the cell viability remained at a high level of 84% (Figure S11, Supporting Information). These results indicate that the fabricated PEDOT:PSS/PVA/Ag NWs film exhibits excellent cytocompatibility and biocompatibility, making it suitable for direct contact with the skin in wearable devices.^[^ ^21^ ^]^

### 2.3 Electromechanical and Electrochemical Performance of Kirigami‐Structured PEDOT:PSS/PVA/Ag NWs/PU Electrodes

To ensure optimal adherence to the skin during movement, it is essential for epidermal devices to be stretchable and elastic. To achieve this, we employed a combination of Kirigami structure and PU encapsulation. The stretchability of stretchable electronics is influenced by various Kirigami patterns, such as uniaxial, biaxial, and square spiral patterns.^[^ ^21^ ^]^ Uniaxial patterns with a pattern of parallel slits are commonly used.^[^ ^44^, ^45^ ^]^ Moreover, the dimensions of Kirigami structures play a significant impact on stretchability, sensitivity, and durability of stretchable electronics. Because the PEDOT:PSS/PVA/Ag NWs film is relatively thin, the primary consideration becomes durability, leading to the adoption of specific dimensions for the Kirigami structures based on previous studies.^[^ ^46^ ^]^ The as‐prepared PEDOT:PSS/PVA/Ag NWs film was transferred to a transfer paper, which was then folded as the required parameters and periodically cut into the Kirigami pattern with a scissor. Afterward, a challenge arises when directly transferring the Kirigami‐structured PEDOT:PSS/PVA/Ag NWs film (KPPA film) onto the PU tape. During stretching, Kirigami structure unfolds on the PU tape and the separation of skeletons within the Kirigami‐structured PEDOT:PSS/PVA/Ag NWs/PU electrodes leads to the exposure of the insulated PU tape (Figure S12, Supporting Information). This presents an issue as it compromises the functionality and reliability of the electrodes. To address this issue, a method was employed to mitigate the phenomenon. Both materials were first stretched before being bonded together. It is important to note that the PU tape has a greater elongation capability compared to the Kirigami‐structured PEDOT:PSS/PVA/Ag NWs film.

**Figure 3:**
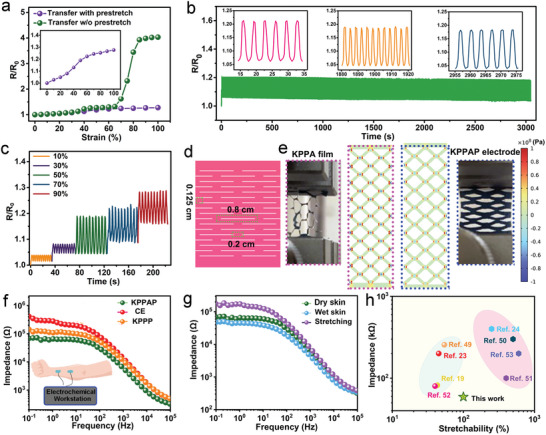
Electromechanical and electrochemical properties of Kirigami‐structured PEDOT:PSS/PVA/Ag NWs/PU electrodes. a) Comparison of the relative resistance of Kirigami‐structured PEDOT:PSS/PVA/Ag NWs/PU electrodes fabricated with prestretch process (PU tape: 26% strain, KPPA film: 16% strain) and without prestretch process. b) Resistance changes of our designed electrode during 1000 cycle of stretch‐release process under 50% strain. c) Resistance changes of our designed electrodes during cyclic stretch‐release processes under different strains. d) Schematic of Kirigami structured PEDOT:PSS/PVA/Ag NWs film. e) Simulated stress distributions and corresponding images of Kirigami‐structured PEDOT:PSS/PVA/Ag NWs (KPPA) film (marked with pink dashed lines) and Kirigami‐structured PEDOT:PSS/PVA/Ag NWs/PU (KPPAP) electrodes (marked with blue dashed lines) under tensile strain. f) Interfacial impedance of different electrode–skin systems from 0.1 to 10^5^ Hz. g) Interfacial impedance of Kirigami‐structured PEDOT:PSS/PVA/Ag NWs/PU electrode–skin systems with different conditions. h) Comparison of interfacial impedance and stretchability of our designed electrode with the reported values.^[^ ^19^, ^23^, ^24^, ^49^, ^50^, ^51^, ^52^, ^53^ ^]^

**Figure** **3**a demonstrates the relative resistance changes of the Kirigami‐structured PEDOT:PSS/PVA/Ag NWs/PU (KPPAP) electrodes fabricated with the prestretch process (PU tape: 26% strain, Kirigami‐structured PEDOT:PSS/PVA/Ag NWs film: 16% strain) and without the prestretch process. Compared with the 3.03 of relative resistance change at 100% strain, the prestretch process endows the Kirigami‐structured PEDOT:PSS/PVA/Ag NWs/PU electrodes with less change in electrical resistance (only 0.28 of resistance change at 100% strain) and stronger resistance to external interference owing to the generated overlapped PEDOT:PSS/PVA/Ag NWs strips on PU tape (Figure S13, Supporting Information). Moreover, the indistinct change in LED brightness visually confirms the slight alteration in electrical resistance of Kirigami‐structured PEDOT:PSS/PVA/Ag NWs/PU electrodes fabricated by the prestretch process (Figure S13, Supporting Information). The relative resistance changes under different cyclic strains also confirm the superiority of prestretch process in fabricating stretchable electrodes (Figure S13, Supporting Information). In the following part, the Kirigami‐structured PEDOT:PSS/PVA/Ag NWs/PU electrodes refer to electrodes fabricated by the prestretch process. Figure 3c shows the distinguishable resistance response of Kirigami‐structured PEDOT:PSS/PVA/Ag NWs/PU electrodes at different tensile strains of 10%, 30%, 50%, 70%, and 90%, respectively, which changes steadily and slightly, indicating the feasibility of the as‐prepared epidermal electrodes for stretchable electrodes and strain sensors. Moreover, the resistance change under different stretching frequencies is stable with slight changes (Figure S10, Supporting Information). The Kirigami‐structured PEDOT:PSS/PVA/Ag NWs/PU electrode possesses a stable resistance change during 1000 cycles of stretch‐release process at 50% strain, indicating its cyclic stability and durability during long‐time movements (Figure 3b). As shown in Figure 3d, the uniaxial kirigami pattern possesses parallel lines in a rectangular arrangement, where the axial spacing between two nearest cut ends is 0.2 cm, the vertical distance between two adjacent cut lines is 0.125 cm, and the cut length is 0.8 cm. Finite element analysis (FEA) on the Kirigami‐structured PEDOT:PSS/PVA/Ag NWs film and Kirigami‐structured PEDOT:PSS/PVA/Ag NWs/PU electrode was conducted to confirm their strain tolerance (Figure 3e; Figure S14, Supporting Information). The large vertical distance between two adjacent cut lines will bring tighter Kirigami structure and reduce the stretchability, while the large cut length will result in looser Kirigami structure and improve the stretchability (Figure S15, Supporting Information). During the stretching process, each strip is rotated around the nodes due to the parallel and symmetrical cuts, and the whole Kirigami‐structured PEDOT:PSS/PVA/Ag NWs film is stretched along the tensile direction (Figure 3e). As the tensile strain increases, the strip is bent along the tensile direction, and the stress distributed in the Kirigami‐structured PEDOT:PSS/PVA/Ag NWs film increases until the occurrence of fracture. Especially, the sharp cuts bring about stress concentration, resulting in larger stress at the nodes (Figure S14, Supporting Information). Compared with the stress distribution in the Kirigami‐structured PEDOT:PSS/PVA/Ag NWs film, the stress distribution in the Kirigami‐structured PEDOT:PSS/PVA/Ag NWs/PU electrode is more uniform and the stress at the nodes is more moderate when the same strains are applied. A Kirigami‐structured PEDOT:PSS/PVA/Ag NWs/PU electrodeadheres to the forearm skin with substantial wrinkles, whose imprints can be clearly observed due to the excellent adaptation of the as‐prepared epidermal electrode to the grooves of wrinkles. In addition, the as‐prepared epidermal electrode can be stretched and compressed on the skin without delamination, and completely recovered (Figure S16, Supporting Information). These above‐mentioned results successfully confirm the superiority of Kirigami structure and PU encapsulation in improving stretchability, conductivity and adhesion to the skin, which are important for the Kirigami‐structured PEDOT:PSS/PVA/Ag NWs/PU electrodes in long‐term human motion monitoring and biopotential recording with high fidelity.

In addition to the above‐mentioned electrochemical properties, low contact impedance is highly desired for epidermal electrodes to collect bioelectrical signals with a high signal‐to‐noise ratio (SNR).^[^ ^47^ ^]^ Figure 3f shows the interfacial impedance of the electrode–skin system from 0.1 to 10^5^ Hz. The skin interfacial impedance spectrum of the Kirigami‐structured PEDOT:PSS/PVA/Ag NWs/PU (KPPAP) electrode exhibits lower values than those of commercial Ag/AgCl gel electrode and kirigami‐structured PEDOT:PSS/PVA/PU (KPPP) electrode over the testing frequency ranges, indicating that the low interfacial impedance has been achieved through the coupling effect of PEDOT:PSS and Ag NWs. The impedance of the Kirigami‐structured PEDOT:PSS/PVA/Ag NWs/PU electrode–skin system at 10 and 100 Hz, which are the median of dominating frequencies for ECG and EMG, are ≈59.76 and 27.41 kΩ, respectively. However, the impedance of the commercial electrode (CE)–skin system significantly increases at 10 and 100 Hz, reaching 191.14 and 71.97 kΩ, respectively. Moreover, it is important to consider the practical working environment of epidermal bioelectrodes (e.g., deformed or sweating states), which can change the states of the electrode–skin system. For example, the sweat film reduces electrode–skin adhesion and causes the detachment of electrodes from skin, resulting in fake signals. Therefore, the interfacial impedance of the Kirigami‐structured PEDOT:PSS/PVA/Ag NWs/PU electrode–skin system under different conditions was investigated (Figure 3g). To simulate the sweat environment, physiological saline is sprayed onto the forearm before the fixation of electrodes. The existence of “sweat” enables the Kirigami‐structured PEDOT:PSS/PVA/Ag NWs/PU electrode–skin system to possess lower interfacial impedance (38.09 kΩ at 10 Hz and 17.19 kΩ at 100 Hz) (Figure S17, Supporting Information). Owing to high adhesion between electrode and skin, the sweat film does not reduce the conformal properties, but enhances ion current conduction efficiency due to the presence of numerous ion charges in saline.^[^ ^48^ ^]^ When the skin is stretched, commercial electrodes are not deformed accompanying the skin because of their rigidity, resulting in the gap between electrodes and skin, or even partial separation from the skin. Owing to the unique Kirigami‐structured, micron thickness and excellent adhesion, the as‐prepared epidermal electrodes can maintain consistent contact areas with the skin upon deformation, resulting in a slight increase in the contact impedance (Figure 3g). However, the Kirigami‐structured PEDOT:PSS/PVA/Ag NWs/PU electrodes still exhibit lower impedance than the commercial electrode when the deformation reaches 25%, which approaches the stretchability of human skin (≈30%). For example, the contact impedance of the stretched Kirigami‐structured PEDOT:PSS/PVA/Ag NWs/PU electrode–skin system with 25% strain at 10 Hz is 123.32 kΩ, which is lower than that of commercial electrodes (191.14 kΩ), while the contact impedance at 100 Hz is only 40.85 kΩ, which is still lower than that of commercial electrodes (71.97 kΩ) (Figure S17, Supporting Information). To demonstrate the superiority of the as‐prepared Kirigami‐structured PEDOT:PSS/PVA/Ag NWs/PU epidermal electrodes, we plot a map comprising of stretchability and interfacial impedance (Figure 3h; Table S1, Supporting Information). These comparisons confirm that our designed electrodes can provide excellent electrochemical performance and low interfacial impedance via the synergistic effects of the unique Kirigami structure, efficient ion‐electron dual conduction paths, and PU encapsulation, which is vital for collecting and transmitting high‐quality biopotential signals. Specifically, the PEDOT:PSS and Ag NWs in the Kirigami‐structured PEDOT:PSS/PVA/Ag NWs/PU electrodes can afford dual channels for both ion and electron transport, enabling more efficient conduction and acquisition of bioelectric signals. In addition, high stretchability resulted from the Kirigami structure, along with strong adhesion between the our designed electrodes and skin, enhances excellent compliance, improving the induced charge density at the bioelectronic interface even under deformation. Moreover, PEDOT: PSS conductive layer can absorb sweat, facilitating the storage of more ions owing to the high polar groups. By capitalizing on these synergistic effects, we have achieved a low interfacial impedance through the rational design of Kirigami‐structured PEDOT:PSS/PVA/Ag NWs/PU electrodes. More importantly, these commercially available materials and facile design of Kirigami structure and encapsulation facilitate the industrial development of high‐performance epidermal electrodes.^[^ ^19^, ^23^, ^24^, ^49^, ^50^, ^51^, ^52^, ^53^ ^]^

### 2.4 Application of the Kirigami‐Structured PEDOT:PSS/PVA/Ag NWs/PU Electrode in Human Motion Detection

**Figure 4:**
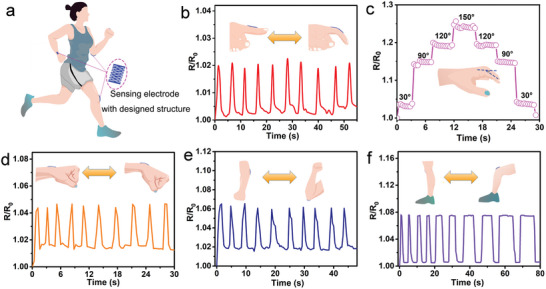
Kirigami‐structured PEDOT:PSS/PVA/Ag NWs/PU electrode‐based strain sensors for human motion detection. a) Schematic diagram of mannequin with different human motions when running. b) Detection of finger bending motion. c) Detection of finger bending at different angles. d) Detection of wrist bending motions. e) Detection of elbow bending motions. f) Detection of knee bending motions.

Accurate monitoring of human movements is important for us to adjust our motion behavior and posture, which can keep the muscles and joints in good condition for healthy lives. The as‐prepared epidermal electrodes have high stretchability, excellent conformal adhesion, and distinguishable resistance changes under tensile strain, and possess the capacity of detecting large human motions without motion artifacts due to the conformal contact with the skin even under large deformation. Owing to the simple cutting strategy used in fabricating Kirigami‐structured PEDOT:PSS/PVA/Ag NWs/PU electrodes, the dimension and cutting sizes of the as‐prepared electrodes can be easily adjusted, making them compatible with different applications. The Kirigami‐structured PEDOT:PSS/PVA/Ag NWs/PU electrodes were attached to the skin on different body parts (e.g., finger, wrist, and elbow), and human motions were identified (**Figure** **4**a). The human motion states (e.g., amplitudes, frequencies, and retention times) can be identified by analyzing the recorded curves of the relative resistance versus time. Because the designed electrodes can be stably adhered to the skin, they can respond to the flexion and extension motions without motion artifacts at different motion speeds. For example, while bending and straightening the index finger repeatedly at a fixed angle, repeatable and stable signals were achieved, indicating high robustness (Figure 4b). When the index finger was bent at angles of 30^o^, 90^o^, 120^o^, and 150^o^, respectively, and held fixed for a certain duration, the relative resistance increased proportionally with the bending angles, and remained unchanged at a fixed angle. During the extensional process, the relative resistance decreases proportionally as the bending angles decrease, with the values nearly identical to those observed during the bending process. These stable changes in resistance indicate a fast response and high robustness (Figure 4c). Similarly, the repeatable and stable signals of the designed electrodes responding to the wrist bending, elbow bending, and knee bending motions, can be identified through analyzing the recorded signal curves, respectively (Figure 4d–f). Interestingly, a larger relative resistance change is observed in knee bending process because of the larger deformation from the leg motion, which results in the separation and contact of the conductive beams in the Kirigami‐structured PEDOT:PSS/PVA/Ag NWs/PU electrodes. Moreover, the changes in resistance during small‐scaled movements, such as exhalation and swallowing, were monitored, and these changes were found to be repeatable and stable (Figure S18, Supporting Information). These human tests confirm the feasibility of our designed electrodes to provide accurate feedback for human motions, and the designability and simple fabrication offer promising potential for stretchable electronics.

### 2.5 Application of the Kirigami‐Structured PEDOT:PSS/PVA/Ag NWs/PU Electrodes in Biopotential Signal Recording

**Figure 5:**
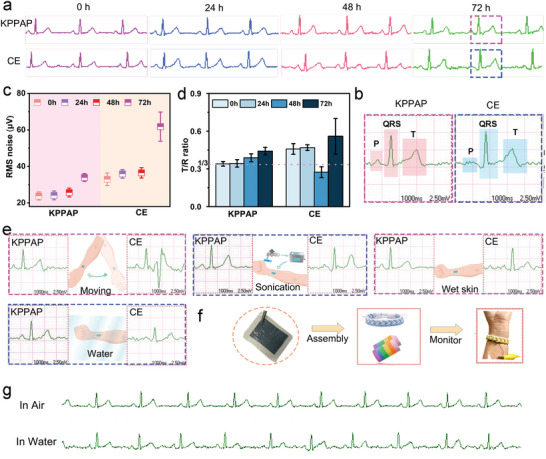
Behaviors of Kirigami‐structured PEDOT:PSS/PVA/Ag NWs/PU electrodes for ECG signal recording. a) Long‐term ECG signal recording at different time using our designed electrodes and commercial electrodes. b) Magnified images of ECG signals marked with the squares in curve (a). c) The RMS noise calculated from the baseline between T and P waves of ECG signals picked by our electrodes and commercial electrodes during ECG recording at 0, 24, 48, and 72 h. d) T/R peak ratio of ECG signals extracted from curve (a). e) ECG signals recorded using Kirigami‐structured PEDOT:PSS/PVA/Ag NWs/PU electrodes and commercial electrodes under different circumstances. f) Schematic of assembling smart wristband for monitoring ECG signals. g) ECG signals recorded using smart wristbands in air and water environment.

Because of high conductivity, low interfacial impedance, and high charge storage capacity, the Kirigami‐structured PEDOT:PSS/PVA/Ag NWs/PU electrodes are used as wearable dry electrodes to detect electrophysiological signals. To demonstrate the excellent performance of our designed electrodes, they are employed to collect biopotential signals (e.g., ECG, EMG, and EOG) with commercial Ag/AgCl gel electrodes as a control group. To record ECG signals, a Kirigami‐structured PEDOT:PSS/PVA/Ag NWs/PU electrode or commercial gel electrode pairs with an area of 3 cm^2^ were placed symmetrically on the inner wrists of the left and right arms of the volunteers, and another designed electrode or commercial gel electrode was attached to the left ankle as the ground electrode. ECG signals were collected intermittently for 72 h in air (**Figure** **5**a; Figure S19, Supporting Information). Three main parameters (P wave, QRS complex, and T wave) can be clearly identified, which are important in clinical diagnosis. For example, the missing of P wave is related to atrial fibrillation, and poor quality of the collected ECG signals may result in a false diagnosis. As shown in Figure 5b, the P wave collected by commercial electrodes is hardly identified after 72 h, while the P wave collected by our designed electrodes is still identified clearly. The noise level of the ECG signals is a deviation of the baseline between T and P waves, and indicates the fluctuations of the signals over time, which is calculated by the root‐mean‐squared (RMS) method (Figure 5c).^[^ ^19^ ^]^ The noise amplitude picked by our designed electrodes is ≈23 µV, which is significantly lower than that of commercial electrodes (32 µV). Moreover, the noise amplitude increases as the monitoring time increases. Specifically, the noise level increase to 35 µV after 72 h, which is only 1/2 of that collected by commercial electrodes (65 µV) owing to high stability of conformability and interfacial impedance of the Kirigami‐structured PEDOT:PSS/PVA/Ag NWs/PU electrodes. Furthermore, the quality of the ECG signals can be assessed by the ratio of T and R wave peak values (T/R ratio), and is close to 1/3, indicating good quality of collected ECG signals.^[^ ^54^ ^]^ The T/R ratios of the ECG signals collected by our designed electrodes are close to 1/3 and fluctuate slightly at different times (Figure 5d), while the T/R ratios picked by the commercial electrodes are relatively far from 1/3 and change significantly with time. Compared with the Kirigami‐structured PEDOT:PSS/PVA/Ag NWs/PU electrodes, the dehydration of the commercial Ag/AgCl gel electrodes strongly influenced the quality of the monitored ECG signals (Figure S20, Supporting Information). As the storage time increases at room temperature, the gel loses more water, resulting in higher interfacial impedance and a deterioration in the quality of ECG signals. Apart from the high‐quality monitoring under static status, the Kirigami‐structured PEDOT:PSS/PVA/Ag NWs/PU electrodes can collect high‐quality ECG signals under extreme conditions (Figure 5e; Figures S21 and S22, Supporting Information). When swinging the hands, the ECG signals recorded by the Kirigami‐structured PEDOT:PSS/PVA/Ag NWs/PU electrodes still possess distinguishable PQRST waveform and the noise level increases slightly, while the ECG signals collected by the commercial electrodes have large fluctuations and inconspicuous PQRST waveform (Figure S23, Supporting Information). Similar phenomenon is observed in the ECG signals collected during writing (Figure S22, Supporting Information). These differences in motion artifacts confirm the conformal contact of our designed electrodes with the skin during human motions owing to the high stretchability and adhesion. In addition, the ECG signal on wet skin is almost the same as that on dry skin with high quality and stability. Even after serious treatments of water rinsing and ultrasonic washing, the Kirigami‐structured PEDOT:PSS/PVA/Ag NWs electrodes can still collect high‐quality ECG signals with distinguishable PQRST waveform, while the disappearance of P wave and large fluctuations are clearly seen in the ECG signals collected by the commercial electrodes. Importantly, the Kirigami‐structured PEDOT:PSS/PVA/Ag NWs/PU electrodes are used for underwater recording of ECG signals (Figure 5e; Figure S21, Supporting Information). ECG signals were recorded after the arms were immersed in water, removed from water, and dried (Figure S22, Supporting Information). Compared with the commercial electrodes, our designed electrodes can collect stable and high‐quality underwater ECG signals owing to constant tight contact with skin in water enabled by the waterproof function and high adhesion of PU tape (Figure S23, Supporting Information). Owing to the feasible tailorability of our designed electrodes, it can be integrated with a wristband or bracelet by adhesion or sewing, which can be worn on the arm for long‐term ECG recording (Figure 5f). As shown in Figure 5j, the smart wristband can collect ECG signals with distinguishable PQRST waveforms in air or water. The ECG signal is also collected by the wristband after exercise, which reflects heart rate change after exercise (Figure S22, Supporting Information). These results suggest the superiority of the Kirigami‐structured PEDOT:PSS/PVA/Ag NWs/PU electrodes in long‐term monitoring of ECG signals with high quality and stability under different extreme conditions.

**Figure 6:**
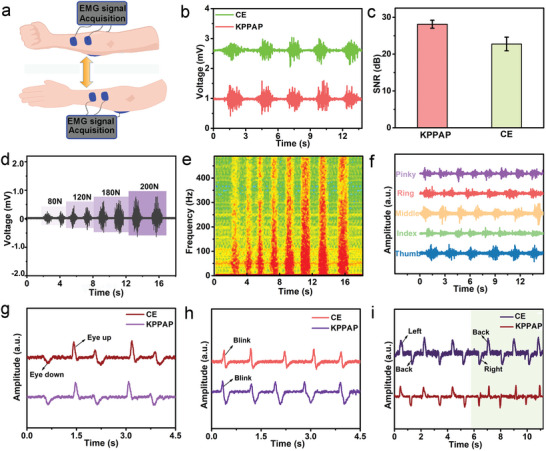
EMG and EOG signals recorded using Kirigami‐structured PEDOT:PSS/PVA/Ag NWs/PU electrodes and commercial electrodes. a) Schematic of recording EMG signals while gripping forces. b) Comparison of EMG signals recorded using Kirigami‐structured PEDOT:PSS/PVA/Ag NWs/PU electrodes and commercial Ag/AgCl gel electrodes. c) SNR values of EMG signals extracted in curves (b). d) EMG signals collected by the as‐prepared epidermal electrodes at various gripping force. e) Time‐frequency spectrogram of the EMG pulse recorded using the as‐prepared epidermal electrodes. f) EMG signals acquired by the as‐prepared epidermal electrodes from the flexion/ extension of different fingers. g) EOG signals recorded by the as‐prepared epidermal and Ag/AgCl electrodes when eyes turning up and down. h) EOG signals recorded by the as‐prepared epidermal and Ag/AgCl electrodes when eyes blinking. i) EOG signals recorded by the as‐prepared epidermal and Ag/AgCl electrodes when eyes turning left and right.

Similarly, the fabricated Kirigami‐structured PEDOT:PSS/PVA/Ag NWs/PU electrodes can be used to record EMG signals associated with the action potential generated by the muscle fibers.^[^ ^20^ ^]^ Two designed electrodes with the center‐to‐center distance of 3 cm were attached to the forearms and a reference electrode was attached to the elbow joints (**Figure** **6**a). During repeated fist clenching and relaxing, EMG signals can be clearly distinguished, and the EMG signal amplitude recorded by our electrodes is higher (Figure 6b). The signal‐to‐noise ratio (SNR) value is ≈28.12 dB, which is higher than that of the commercial gel electrode (22.75 dB) (Figure 6c). Moreover, the measured spectra of EMG signals are subjected to fast Fourier transform (FFT) treatment (Figure S24a, Supporting Information). Their frequency distribution is basically the same, but the amplitude of the EMG signal collected by the Kirigami‐structured PEDOT:PSS/PVA/Ag NWs/PU electrodes is significantly higher than that collected by the commercial electrodes. Figure 6d shows the EMG signals at different gripping force levels. When 80, 120, 180, and 200 N of force were applied to the grip strength meter, the peak‐to‐peak amplitude and the signal intensity increased as the gripping force increased (Figure S24b, Supporting Information), which is also reflected by the main energy distribution in the frequency spectrum of EMG signals by short‐time Fourier transform (STFT) treatment (Figure 6e). In addition to the significant motions of muscle fibers, the Kirigami‐structured PEDOT:PSS/PVA/Ag NWs/PU electrodes can record low amplitude EMG signals induced from finger extension or flexion (Figure 6f; Figure S24c, Supporting Information). As shown in Figure 6g–i, commercial electrodes and our designed electrodes are used to record eye potential, which can reflect eye movements, such as blinking and vertical/horizontal movements. Accompanied with unambiguous eye movements, EOG signals with specific characteristics were recorded, and the signals recorded by our designed electrodes possess less noise and larger amplitude than that obtained by commercial electrodes. This superiority is resulted from its intimate contact with the skin during motion, and its high current density and efficiency in capturing the electrical potential. EEG recording provides a noninvasive method to monitor brain activity, but EEG signal amplitude is relatively low, bringing about some challenges in recording EEG signals. Figure S24d (Supporting Information) shows the collected EEG signals with significant differences in the time domain during eye opening and closing. After the FFT treatment, strong frequency bands in the power spectral density (PSD) of the alpha rhythm (8–13 Hz) and beta rhythm (14–30 Hz) are observed during eye closing, as we expect that thinking is associated with high‐frequency brain activity (Figure S24d, Supporting Information).^[^ ^21^ ^]^

### 2.6 Application of the Kirigami‐Structured PEDOT:PSS/PVA/Ag NWs/PU Electrodes in Human–Machine Interaction

**Figure 7:**
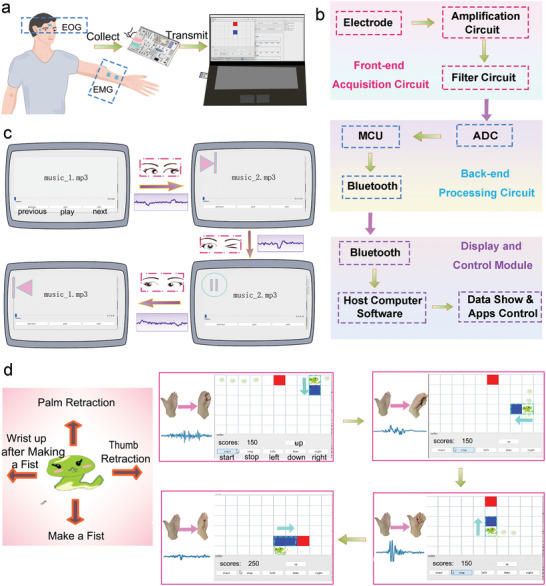
Applications of the Kirigami‐structured PEDOT:PSS/PVA/Ag NWs/PU electrodes in HMI. a) Schematic illustration of the as‐prepared epidermal electrodes for applications in the operations of music play/switch and snake game using EOG and EMG signals. b) Schematic diagram showing the working process of our designed electrodes to collect bioelectric signals for human–computer interaction. c) Demonstration of controlling a computer to play/switch music using EOG signals. d) Demonstration of playing snake game using EMG signals.

The HMI based on bioelectrical signals holds immense importance in the field of AI. The capability to capture high‐quality and consistent biopotential signals makes the Kirigami‐structured PEDOT:PSS/PVA/Ag NWs/PU electrodes suitable for HMI applications. One notable example is the utilization of the Kirigami‐structured PEDOT:PSS/PVA/Ag NWs/PU electrodes to record EOG signals associated with eye movements. These signals can be employed for synchronized operations such as music play/switch, thereby assisting individuals with disabilities in performing basic tasks. Additionally, the EMG signals captured from hand motions can be effectively employed to enable synchronous gameplay of the snake game (**Figure** **7**a).^[^ ^55^, ^56^ ^]^ By utilizing distinguishable biopotential signals captured through our electrodes, a control system based on human activity has been developed. The system's process of collecting bioelectric signals for human‐computer interaction is illustrated in Figure 7b. At the outset, the Kirigami‐structured PEDOT:PSS/PVA/Ag NWs/PU electrodes are adhered to specific locations on the human body to capture EMG or EOG signals. After amplification and filtering processes, these collected EOG or EMG signals are transmitted and converted to digital information via analog‐to‐digital converter (ADC). These received signals are transformed into control commands in real time by the microprogrammed controller unit (MCU) module, which is sent to the computer through bluetooth for manipulating the music play/switch and snake game in our home‐made software (Figure S25, Supporting Information). For the EOG‐based HMI, diverse motions of nictation, looking right, and looking left are employed to control the music play/switch (Figure 7c). Specifically, the nictation motion of eyes corresponds to pausing playback, eye motion of looking right corresponds to switching to the next song, while eye motion of looking left corresponds to switching to the previous song. In Figure 7c and Movie S1 (Supporting Information), the EOG signals collected through our electrodes can accurately and continuously achieve arbitrary music switch/play.

Similarly, four distinguishable types of gestures (palm retraction, thumb retraction, make a fist, and wrist up after making a fist) are introduced into a gesture‐controlled greedy snake game that manipulates the movement of small snakes (blue squares) to eat food (red squares). It can be observed that the snake game is effectively controlled by predefined gestures accompanied by distinct electromyography signals, resulting in a satisfying gaming experience (Figure 7d; Movie S2, Supporting Information). When performing the gestures for the left, right, up, and down movements, the blue tile in the game promptly responds in real‐time, showcasing both high sensitivity and accuracy without noticeable delays. These outcomes demonstrate the practicality of employing the Kirigami‐structured PEDOT:PSS/PVA/Ag NWs/PU electrodes in stretchable electronics and HMI applications.

## 3 Conclusion

We have developed a highly stretchable, low interfacial impedance, and skin‐conformal epidermal electrode for long‐term biopotential monitoring and HMI by leveraging the ionic/electronic current from the dual conductive network of PEDOT:PSS/Ag NWs, incorporating a Kirigami structure, and utilizing unique encapsulation. The resulting epidermal electrodes demonstrate a low sheet resistance of ≈3.9 Ω sq^−1^, a large skin‐compliant stretchability of over 100%, sufficient mechanoelectrical stability, good biocompatibility, and a low interfacial impedance of ≈27.41 kΩ at 100 Hz and 59.76 kΩ at 10 Hz.

Compared to existing dry electrodes or standard gel electrodes, our prepared electrodes possess comparable interfacial impedance and stretchability, and can reliably acquire long‐term, high‐quality, and stable ECG signals even under extreme conditions, such as sweat, wetness, and movement. This is due to their high intimate conformability and waterproof ability. Additionally, the Kirigami‐structured PEDOT:PSS/PVA/Ag NWs/PU electrodes enable the collection of high‐quality and distinguishable EMG, EOG, and EEG signals, thanks to their intimate contact with the skin and low contact impedance. The remarkable ability to record high‐quality and stable biopotential signals allows our designed electrodes to manipulate music play/switch and play the snake game with high sensitivity and accuracy without noticeable delays. Our proposed advanced materials and structures for designing high‐performance epidermal electrodes offer a unique perspective for epidermal electronics.

## 4 Experimental Section

### Materials

PEDOT:PSS aqueous solution (Clevios PH 1000) with 1.3 wt.% of PEDOT: PSS was purchased from Heraeus Corporation. Sodium chloride (NaCl, purity: ≥99.5%) and silver nitrate (AgNO_3_, purity: ≥99.8%) were purchased from Sinopharm Chemical Reagent Co., Ltd. PVA (*M*_w_≈145000), polyvinylpyrrolidone (PVP, *M*_w_≈1300000), and DMSO (purity: ≥99.9%) were purchased from Shanghai Aladdin Biochemical Technology Co., Ltd. Ethylene glycol (purity: >99%) was purchased from Shanghai Macklin Biochemical Co., Ltd. All the chemicals were used as received without further purification. Waterproof PU tape was purchased from Taobao.com. Commercial electrode was purchased from Hangzhou Schindler Radio Equipment Co., Ltd., the metal electrode core diameter was 10 mm, and the gel diameter was ≈16 mm.

### Synthesis of Ag NWs

Ag NWs were synthesized by a polyol reduction method. Specifically, PVP was first dissolved in EG, achieving a concentration of 12.5 mg mL^−1^. Then, 2 mL of NaCl/EG solution (0.42 mg mL^−1^) was added to the PVP/EG solution. After 3 min of vigorous stirring, 5 mL of AgNO_3_/EG solution (50 mg mL^−1^) was added into the above‐mentioned solution, stirred vigorously for another 5 min, and heated at 170 °C for 45 min. After the finishing of the reaction, it was quickly cooled in an ice bath, and was successively washed once with deionized water and anhydrous ethanol for three times.

### Fabrication of PEDOT:PSS/PVA/Ag NWs Conductive Films

To improve the conductivity of PEDOT:PSS, PEDOT: PSS was doped by DMSO. Sample nomenclature was shown in Table S2 (Supporting Information). Specifically, 5 g of PEDOT:PSS aqueous solution was co‐mixed with 0.15 g of DMSO, and then the treated PEDOT: PSS solution was thoroughly mixed with different amounts of PVA aqueous solution (5 wt.%). PEDOT: PSS films with a thickness of ≈3–4 µm were fabricated by casting the PEDOT:PSS/DMSO/PVA solution onto a glass plate, which was cured at 70 °C for 30 min. Subsequently, Ag NWs/ethanol solution was sprayed onto the as‐prepared PEDOT:PSS/PVA film on a glass plate with a consistent spraying rate of 12 µL s^−1^ and a distance of 9 cm between the nozzle and the substrate. After continuous spraying for 60 s, 50 s of interval was required before the following spraying. To accelerate the evaporation of ethanol and improve the adhesion between Ag NWs and PEDOT:PSS/PVA film, heating treatment at 85 °C was required to facilitate the combination of PEDOT:PSS/PVA film and Ag NWs, avoiding the detachment of Ag NWs during applications owing to the partial embedment of Ag NWs in the PEDOT:PSS/PVA film, yielding a PEDOT:PSS/PVA/Ag NWs film on a glass plate.

### Fabrication of the Kirigami‐Structured PEDOT:PSS/PVA/Ag NWs/PU Electrodes

The as‐prepared PEDOT:PSS/PVA/Ag NWs films were transferred to a transfer paper, which was folded and periodically cut into the designed pattern with a scissor. After unfolding and flattening, the kirigami structure was designed on PEDOT:PSS/PVA/Ag NWs film. The as‐prepared Kirigami‐structured PEDOT:PSS/PVA/Ag NWs film on transfer paper was pre‐stretched to 16% strain, which was attached to the waterproof PU film with a tensile strain of 26%. Due to the adhesion difference, the Kirigami‐structured PEDOT:PSS/PVA/Ag NWs film was transferred to the PU tape, yielding a Kirigami‐structured PEDOT:PSS/PVA/Ag NWs/PU electrode. Similarly, the PEDOT:PSS/PVA/Ag NWs film on transfer paper without pre‐stretching was transferred to the non‐stretched PU tape, yielding the other kind of Kirigami‐structured PEDOT:PSS/PVA/Ag NWs/PU electrode.

### Material Characterization

The surface morphology of the samples were characterized using a field‐emission scanning electron microscopy (FE‐SEM, Nova Nano SEM 450 USA). The chemical properties and valence states were conducted on an X‐ray photoelectron spectroscopy (XPS ESCALAB 250Xi, Thermo, USA) with an exciting line of Al Kα. The Raman spectra were collected on a confocal laser micro Raman spectrometer with 532 nm excitation (HORIBA Jobin‐Yvon, France). The sheet resistances were tested using a four‐probe sheet resistance meter (HPS2524, HELPRSS, China). The stress‐strain curves were collected using an electronic universal testing machine. The electrical and mechanical properties were calculated from five samples, and standard deviation calculations were performed using Origin software. In terms of the sheet resistance, the measured points of sheet resistance were shown in Figure S26 (Supporting Information). The electromechanical performances were conducted using digital multimeter (RIGOL DM 3068) and electronic universal testing machine (ZQ‐990LB, ZhiQu Co. Ltd., China).

### Cell Culture to Conform Biocompatibility

The in vitro cytotoxity of PEDOT:PSS/PVA/Ag NWs films was tested using NIH 3T3 fibroblasts. Specifically, the PEDOT:PSS/PVA/Ag NWs films were washed with ethanol and phosphate buffered salts (PBS). Then, the cell suspension with 2×10^4^ cells was inoculated on PEDOT:PSS/PVA/Ag NWs films placed in culture plates. After the introduction of fresh medium comprising of 89% Dulbecco's modified eagle medium (DMEM, GibcoTM, high glucose), 10% fetal bovine serum (Gibco, qualified, Brazil), and 1% Penicillin‐Streptomycin (Gibco, 10 000 U mL^−1^) in volume fraction, culture plates were placed in an incubator with 5% CO_2_ at 37 °C. The live/dead cell assays were performed to calculate cell viability on day 1 and day 3. Three parallel samples were extracted, then the mean and standard deviation were calculated using Origin software. Cells were observed using a fluorescence microscope and cell viability was derived from the standardized ratio of the number of living cells to the total number of cells.^[^ ^22^ ^]^

### Electrochemical Characterization

The electrochemical properties of the PEDOT:PSS/PVA and PEDOT:PSS/PVA/Ag NWs film were studied in a three‐electrode system through an electrochemical working station (CHI660E, CH Instrument). The PEDOT:PSS/PVA film or PEDOT:PSS/PVA/Ag NWs film, platinum wire, and commercial Ag/AgCl electrode were employed as working, counter, and reference electrodes, respectively; 0.01 M Dulbecco's phosphate buffered saline (DPBS) was used as an electrolyte. Electrochemical impedance was obtained at an initial potential of 0 V with an AC voltage amplitude of 10 mV in the frequency range of 1–10^5^ Hz. CVs were recorded at different scan rates of 10, 20, 30, 40, and 50 mV s^−1^, with an electrochemical window of 0–0.5 V. Charging current was derived from the CVs, and the specific capacitance of the electrodes was the linear slope of the curves of charging current versus scan rate. CSC was calculated by the cyclic voltammetry method with voltages from 0 to 0.5 V at a scan rate of 50 mV s^−1^. The interfacial impedance of skin/electrode was performed using a pair of same electrodes (e.g., Kirigami‐structured PEDOT:PSS/PVA/Ag NWs/PU, Kirigami‐structured PEDOT:PSS/PVA/PU, and commercial Ag‐AgCl gel electrodes) on an electrochemical working station (CHI660E, CH Instrument) within the frequency range of 0.1–10^5^ Hz. All electrodes had a certain area of 3 cm^2^, and the center‐to‐center distance was 5 cm on forearm skin.

### Electrophysiological Signal Recording

When recording bioelectric signals, metal wires were connected the Ag NWs layer of the epidermal electrodes, realizing the conduction of bioelectric signals (Figure S27, Supporting Information). Two working electrodes were placed on the volunteer's forearms and the reference electrode was placed at the ankle of the left foot, which were connected with an ECG meter (Heal Force PC‐80) to collect the ECG signals. Especially for the long‐term ECG signal recording, the Kirigami‐structured PEDOT:PSS/PVA/Ag NWs/PU electrodes and commercial electrodes were adhered to the skin throughout the duration of the recordings, while normal activities such as bathing and exercise were conducted as usual. No other operation, such as the replacement of commercial gel electrodes was conducted during the recording test. The SNR and T/R ratio of ECG signals were calculated from ten samples fabricated at different times, and 20 of PQRST waveform were randomly selected in each ECG signal to calculate the SNR and T/R ratio, and then the average and standard deviation were calculated in Origin software. EMG, EOG, and EEG signals were recorded in triple‐electrode system with a Signal Acquisition and Processing Circuit (SiChiRay). For EMG signals acquisitions from fist clenching, grip strength, and finger movements, two working electrodes with the center‐to‐center distance of 3 cm were attached to the forearms and a reference electrode was attached to the elbow joints. For the EOG signals measurements during the up‐and‐down movements of eyes, two working electrodes were placed on the upper and lower eyelids, respectively, and the reference electrode was placed on the forehead. When recording the EOG signals from side‐to‐side movements of eyes, two working electrodes were placed on the outer canthi of each eye, and the reference electrode was placed at the forehead. For EEG signal collection, two working electrodes were placed on the forehead, and the reference electrode was placed on the neck. Fast Fourier transform and short‐time Fourier transform of the signals were performed to obtain the corresponding spectra.

### Human–Machine Interaction

To control the music play/switch via the EOG signals from the side‐to‐side movements of eyes, two working electrodes were placed on the outer canthi of each eye, and the reference electrode was placed at the forehead. To play the snake game via the EMG signals, two working electrodes with the center‐to‐center distance of 3 cm were attached to the forearms and a reference electrode was attached to the elbow joints. After amplification and filtering processes, these collected EOG or EMG signals were transmitted and converted to digital information via ADC. After data integration and processing by MCU, these signals were sent to the computer through bluetooth, which were processed into control commands to manipulate the music play/switch and Snake Game in the home‐made software written by Python's third‐party library PyQt5.

## Conflict of Interest

The authors declare no conflict of interest.

## Supporting information

Supporting Information

Supporting Information

Supporting Information

## Data Availability

The data that support the findings of this study are available on request from the corresponding author. The data are not publicly available due to privacy or ethical restrictions.
