# Supplementary material for: Kirigami‐Structured, Low‐Impedance, and Skin‐Conformal Electronics for Long‐Term Biopotential Monitoring and Human–Machine Interfaces
Source: Adv Sci (Weinh). 2023 Nov 20;11(1):2304871. doi: 10.1002/advs.202304871 (PMC10767437; doi:10.1002/advs.202304871)
Supplement: Supplementary file 1 — Supporting Information [file ADVS-11-2304871-s003.pdf]

ADVANCED SCIENCE
Open Access

Supporting Information

for *Adv. Sci.*, DOI 10.1002/advs.202304871

Kirigami-Structured, Low-Impedance, and Skin-Conformal Electronics for Long-Term Biopotential Monitoring and Human–Machine Interfaces

Meili Xia, Jianwen Liu, Beom Jin Kim, Yongju Gao, Yunlong Zhou, Yongjing Zhang, Duxia Cao, Songfang Zhao, Yang Li* and Jong-Hyun Ahn**

Supporting Information

Kirigami-structured, low-impedance and skin-conformal electronics for long-term biopotential monitoring and human-machine interfaces

Meili Xia, Jianwen Liu, Beom Jin Kim, Yongju Gao, Yunlong Zhou, Yongjing Zhang, Duxia Cao, Songfang Zhao, Yang Li,* and Jong-Hyun Ahn**

M.L. Xia, Y. L. Zhou, Y. J. Zhang, Prof. D. X. Cao, Prof. S. F. Zhao

School of Materials Science and Engineering, University of Jinan, Jinan 250022, China

E-mail: zhaosongfang@163.com (Prof. S. Zhao)

J.W. Liu, Prof. Y. Li

School of Information Science and Engineering, University of Jinan, Jinan 250022, China

Email: ise_liy@ujn.edu.cn (Prof. Y. Li)

Y.J. Gao

Shandong Zhongke Advanced Technology Co., Ltd., Jinan 250000, China

Prof. Y. Li

School of Microelectronics, Shandong University, Jinan, 250101, China

Email: ise_liy@ujn.edu.cn (Prof. Y. Li)

B.J. Kim, Prof. J.-H. Ahn

School of Electrical and Electronic Engineering, Yonsei University, Seoul 03722, Republic of Korea

Email: ahnj@yonsei.ac.kr (Prof. J. -H. Ahn)

Contents

Figure S1. (a) SEM image of Ag NWs. (b) Top-view SEM image of PEDOT:PSS/PVA film. (c, d) Top-view SEM images of PEDOT:PSS/PVA/Ag NWs film. (e, f) Cross-sectional SEM image of PEDOT:PSS/PVA/Ag NWs film.

Figure S2. (a) Nyquist diagram of the Kirigami-structured PEDOT:PSS/PVA/Ag NWs/PU electrode-skin system, and the experimental values fit well with the simulated values of the contact impedance of electrode-skin system. (b) Measured and simulated contact impedance. These excellent fits indicate the rationality of the equivalent circuit model.

Figure S3. (a, b) XPS spectra of C1s region of the (a) PEDOT:PSS/PVA film and (b) PEDOT:PSS/PVA/Ag NWs film. (c, d) XPS spectra of S2p region of the (c) PEDOT:PSS/PVA film and (d) PEDOT:PSS/PVA/Ag NWs film.

Figure S4. (a) Full XPS spectra of the PEDOT:PSS/PVA side on the PEDOT:PSS/PVA/Ag NWs film. (b) XPS spectra of C1s region. (c) XPS spectra of O1s region. (d) XPS spectra of S2p region.

Figure S5. (a, b) Optical microscope images of Ag NWs network fabricated by spraying Ag NWs solution on PEDOT:PSS/PVA films: (a) with 0% water, (b) with 50% water.

Figure S6. (a) The digital images show that the PEDOT:PSS/PVA films with low PVA loadings are broken while being detached. (b) Stress-strain curves of PEDOT:PSS/PVA films with different PVA loadings.

Figure S7. The surficial morphologies of PEDOT:PSS/PVA/Ag NWs films after experiencing peeling tests by 3M tape. The addition of water can enhance the interfacial interactions between Ag NWs and PEDOT:PSS/PVA films.

Figure S8. Comparison of sheet resistance of PEDOT:PSS/PVA film and PEDOT:PSS/PVA/Ag NWs film.

Figure S9. SEM images of Ag NWs in the PEDOT:PSS/PVA/Ag NWs film.

Figure S10. (a) Phase degree-frequency diagram of PEDOT:PSS/PVA and PEDOT:PSS/PVA/Ag NWs film. (b) CVs of PEDOT:PSS/PVA films as electrodes at different scan rates (0-0.5V). (c, d) CVs of PEDOT:PSS/PVA (c) and PEDOT:PSS/PVA/Ag NWs films (d) at different scan rates in 0.01 M PBS (0-0.8V). (e) CVs of PEDOT:PSS/PVA and PEDOT:PSS/PVA/Ag NWs films as electrodes at a scan rate of 50 mV s^{-1}. (f) Linear relationship between charging current and scan rate for PEDOT:PSS/PVA films and PEDOT:PSS/PVA/Ag NWs films in 0.01 M PBS (0-0.8V).

Figure S11. (a, b) Fluorescent images of stained NIH 3T3 cells cultured on control substrate and PEDOT:PSS/PVA/Ag NWs film after 72h. Live cells: green. Dead cells: red. (c) Cell viability rate at 24 h and 72 h of incubation.

Figure S12. Digital images of Kirigami-structured PEDOT:PSS/PVA/Ag NWs/PU electrodes during stretching.

Figure S13. (a) Digital images of brightness changes of LED light during the stretch-release process of the Kirigami-structured PEDOT:PSS/PVA/Ag NWs/PU electrode. (b, c) Resistance changes of the Kirigami-structured PEDOT:PSS/PVA/Ag NWs/PU electrodes fabricated without prestretch process (b) and with prestretch process (c) at various strains, respectively. Insets are the digital images of the corresponding electrodes. (d) Resistance change of the Kirigami-structured PEDOT:PSS/PVA/Ag NWs/PU electrode under 30% strain at diverse frequencies.

Figure S14. Simulated stress distributions of the Kirigami-structured PEDOT:PSS/PVA/Ag NWs film and Kirigami-structured PEDOT:PSS/PVA/Ag NWs/PU electrode at different strains. When same strains are exerted, Kirigami-structured PEDOT:PSS/PVA/Ag NWs/PU electrodes bear less stress at the fracture points due to the protection of PU tape, and it is more difficult to break, preventing damage to the conductive network.

Figure S15. Stretching processes of Kirigami patterns with different cutting sizes.

Figure S16. Top view of a Kirigami-structured PEDOT:PSS/PVA/Ag NWs/PU electrode on skin with different deformation.

Figure S17. (a) Interfacial impedance of different electrode-skin systems from 0.1 to 10^5 Hz. (b) Comparison of interfacial impedance of different electrode-skin systems in different conditions at 10 Hz and 100 Hz.

Figure S18. Kirigami-structured PEDOT:PSS/PVA/Ag NWs/PU electrode-based strain sensors for (a) detecting cough process, (b) exhale process.

Figure S19. Long-term ECG signal recording at different time using Kirigami-structured PEDOT:PSS/PVA/Ag NWs/PU electrodes and commercial electrodes at 0 h, 24 h, 48 h and 72 h, respectively.

Figure S20. (a) Weight loss percentage of commercial electrodes placed at room temperature for different time. (b) Interfacial impedance of commercial electrodes placed at room temperature for different times. (c) ECG signals recorded with commercial electrodes placed for 72 hours and 0 hours.

Figure S21. ECG signals recorded using Kirigami-structured PEDOT:PSS/PVA/Ag NWs/PU electrodes and commercial electrodes under different circumstances (e.g., moving, wet skin, sonication, and under water).

Figure S22. ECG signals recorded by Kirigami-structured PEDOT:PSS/PVA/Ag NWs/PU electrodes and commercial electrodes after arms are immersed in water, removed from water, and dried, and ECG signals recorded by wristband after exercise.

Figure S23. The RMS noise calculated from the baseline between T and P waves of ECG signals picked by Kirigami-structured PEDOT:PSS/PVA/Ag NWs/PU electrodes and commercial electrodes during ECG recording under different circumstances (e.g., moving, wet skin, sonication, and under water).

Figure S24. (a) Spectra of the EMG pulse recorded using the Kirigami-structured PEDOT:PSS/PVA/Ag NWs/PU electrode and commercial electrodes. (b) Variations of the EMG signal amplitude with different gripping force. (c) EMG signal amplitude produced by the flexion/extension of different fingers. (d) Time-domain EEG signals of open eyes and closed eyes recorded by the Kirigami-structured PEDOT:PSS/PVA/Ag NWs/PU electrode. (e) Spectra of the EEG signal with eyes

open and eyes closed.

Figure S25. (a, b) Layout of circuit with the Kirigami-structured PEDOT:PSS/PVA/Ag NWs/PU electrodes for applications in the operations of music play/switch using EOG signals (a) and snake game using EMG signals (b).

Figure S26. Measured points of sheet resistance on one sample.

Figure S27. Method of metal wires connected to epidermal electrodes.

Table S1. Comparison of our Kirigami-structured PEDOT:PSS/PVA/Ag NWs/PU electrodes with the reported electrodes

Table S2. Sample Naming Explanation

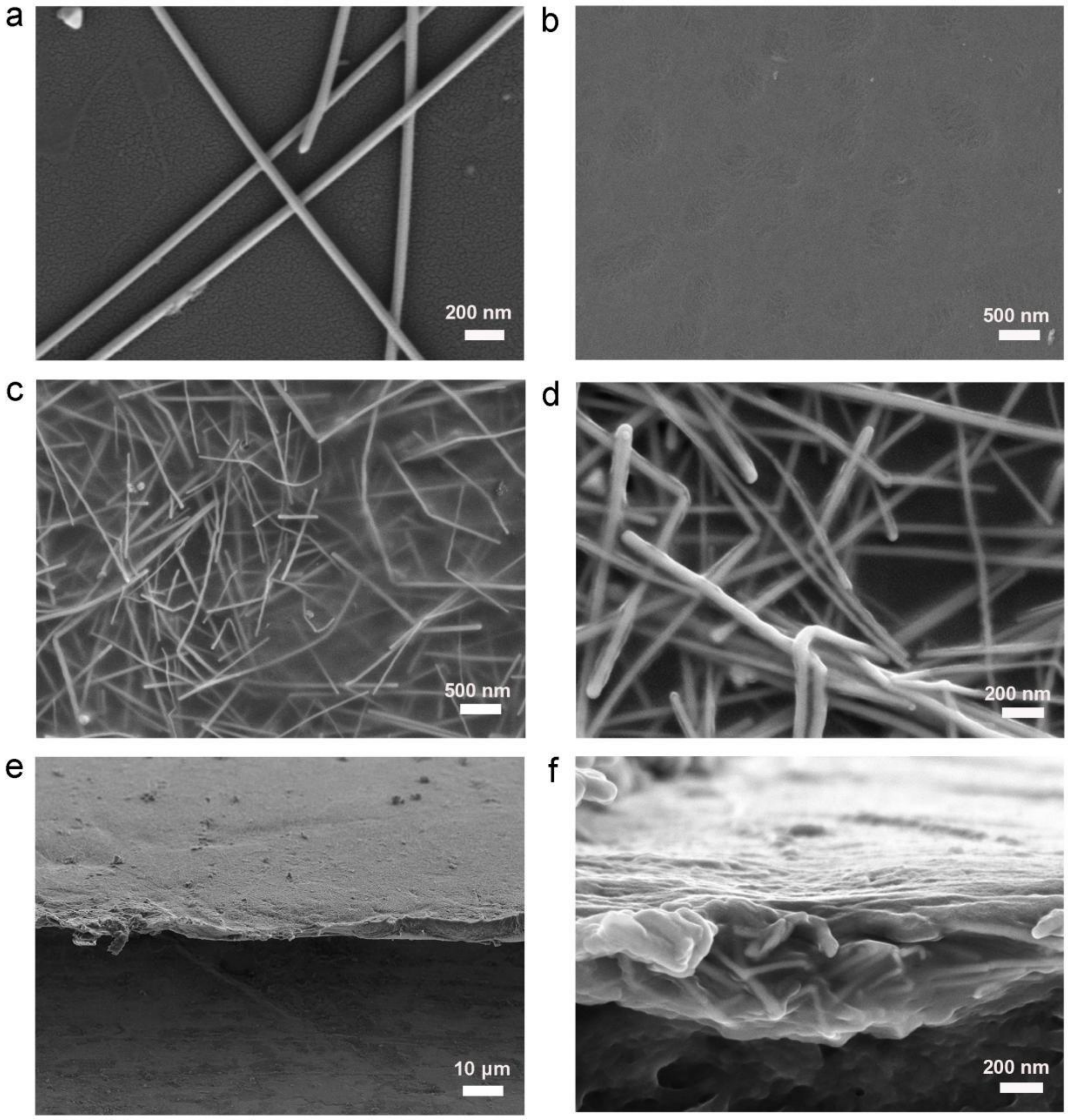

Figure S1. (a) SEM image of Ag NWs. (b) Top-view SEM image of PEDOT:PSS/PVA film. (c, d) Top-view SEM images of PEDOT:PSS/PVA/Ag NWs film. (e, f) Cross-sectional SEM image of PEDOT:PSS/PVA/Ag NWs film.

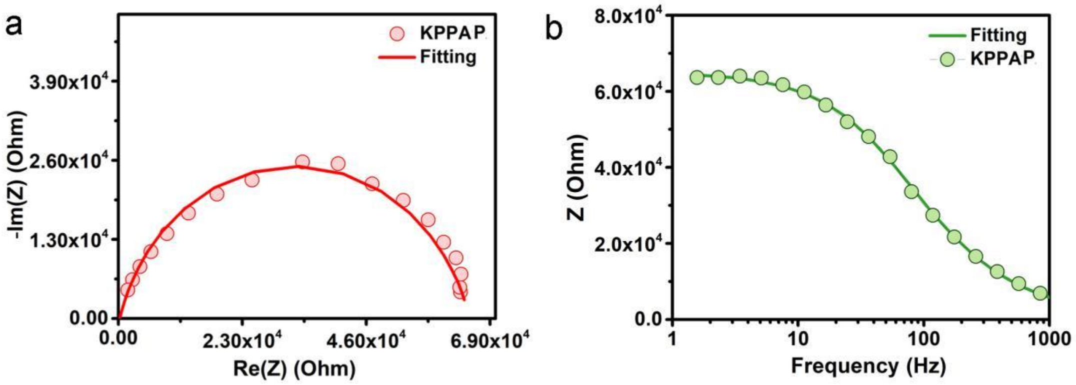

Figure S2. (a) Nyquist diagram of the Kirigami-structured PEDOT:PSS/PVA/Ag NWs/PU electrode-skin system, and the experimental values fit well with the simulated values of the contact impedance of electrode-skin system. (b) Measured and simulated contact impedance. These excellent fits indicate the rationality of the equivalent circuit model.

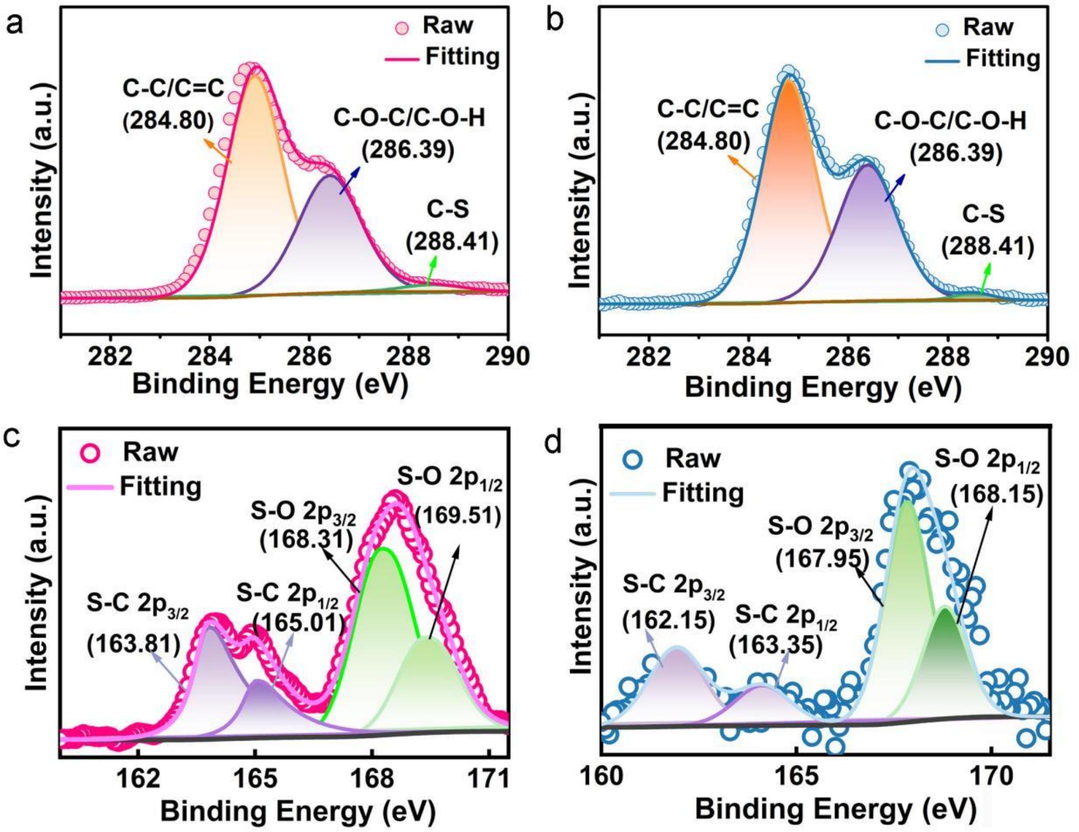

Figure S3. (a, b) XPS spectra of C1s region of the (a) PEDOT:PSS/PVA film and (b) PEDOT:PSS/PVA/Ag NWs film. (c, d) XPS spectra of S2p region of the (c) PEDOT:PSS/PVA film and (d) PEDOT:PSS/PVA/Ag NWs film.

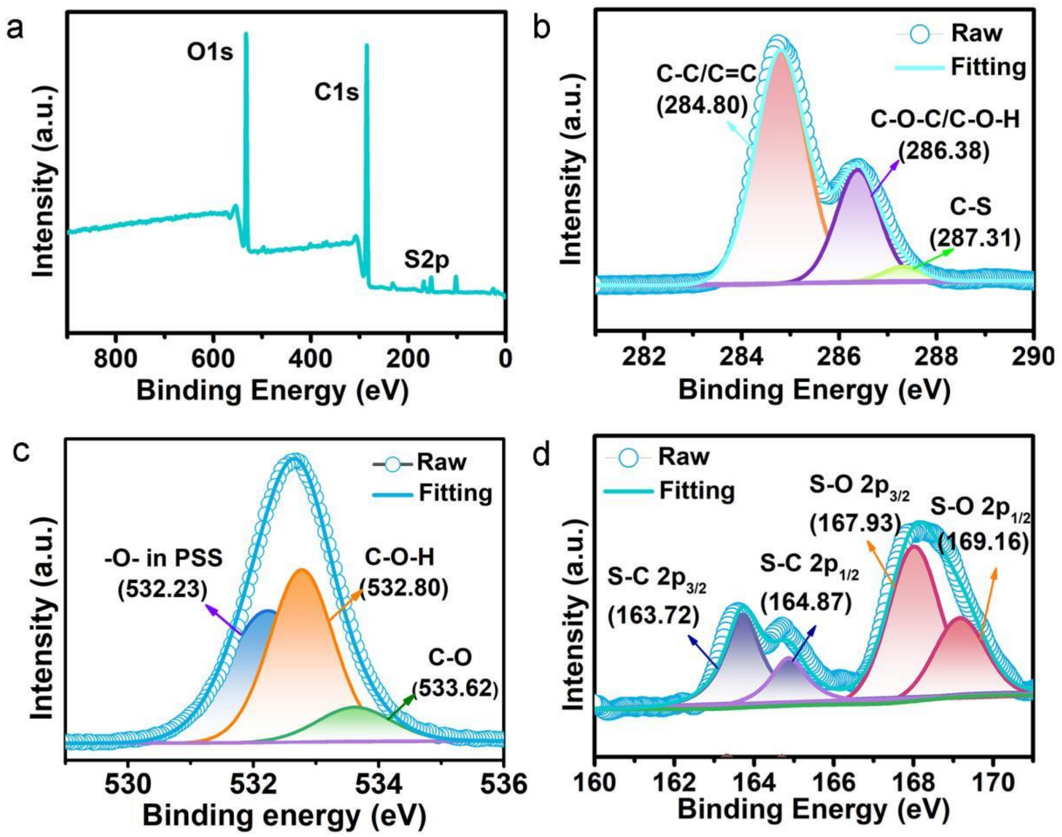

Figure S4. (a) Full XPS spectra of the PEDOT:PSS/PVA side on the PEDOT:PSS/PVA/Ag NWs film. (b) XPS spectra of C1s region. (c) XPS spectra of O1s region. (d) XPS spectra of S2p region.

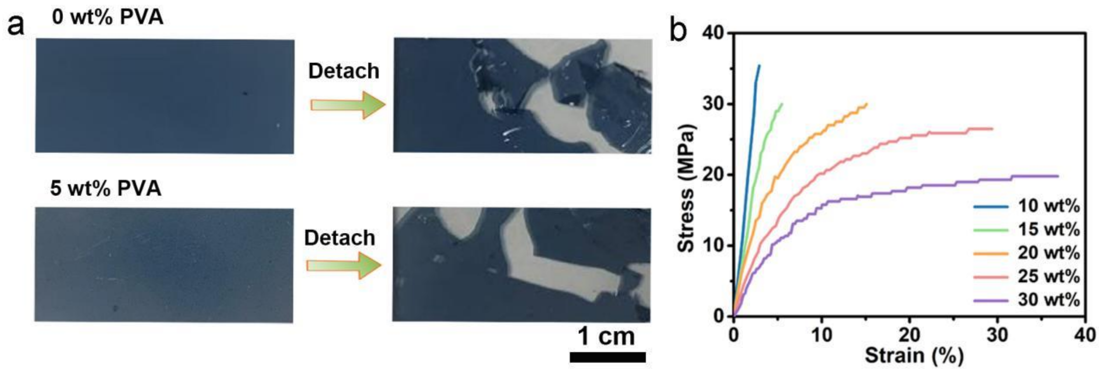

Figure S5. (a, b) Optical microscope images of Ag NWs network fabricated by spraying Ag NWs solution on PEDOT:PSS/PVA films: (a) with 0% water, (b) with 50% water.

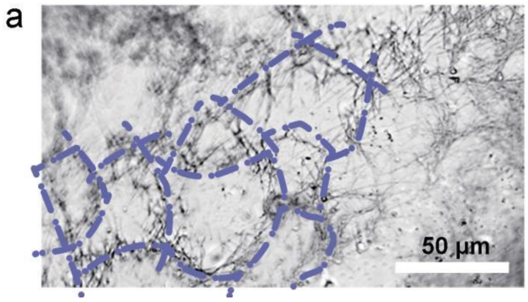

Figure S6. (a) The digital images show that the PEDOT:PSS/PVA films with low PVA loadings are broken while being detached. (b) Stress-strain curves of PEDOT:PSS/PVA films with different PVA loadings.

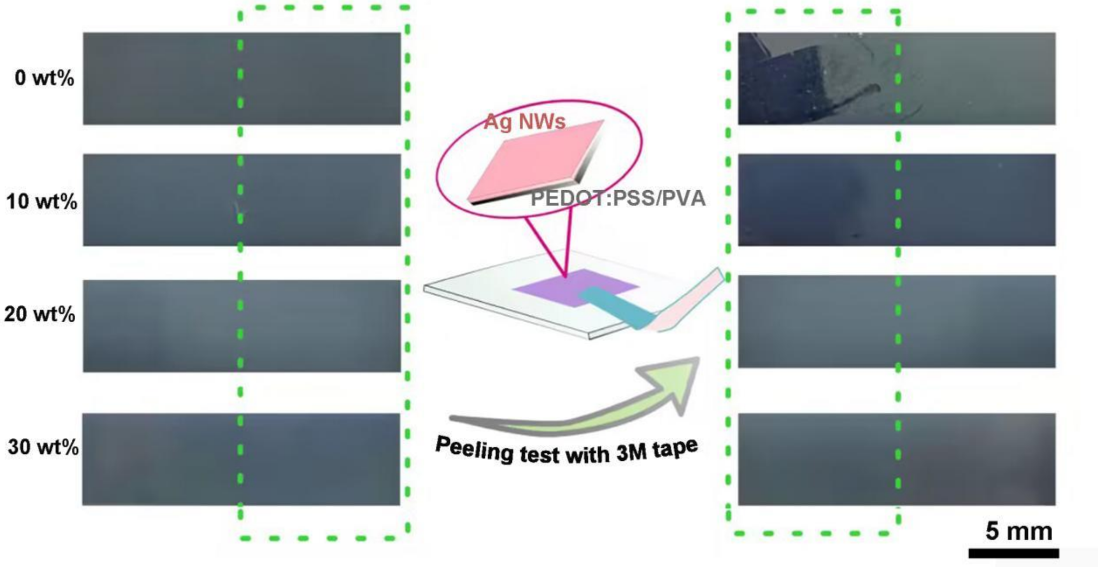

Figure S7. The surficial morphologies of PEDOT:PSS/PVA/Ag NWs films after experiencing peeling tests by 3M tape. The addition of water can enhance the interfacial interactions between Ag NWs and PEDOT:PSS/PVA films.

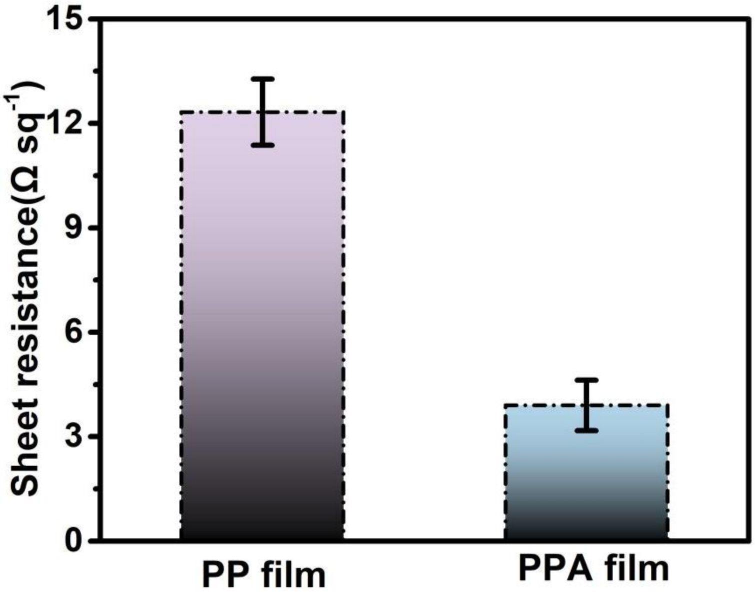

Figure S8. Comparison of sheet resistance of PEDOT:PSS/PVA film and PEDOT:PSS/PVA/Ag NWs film.

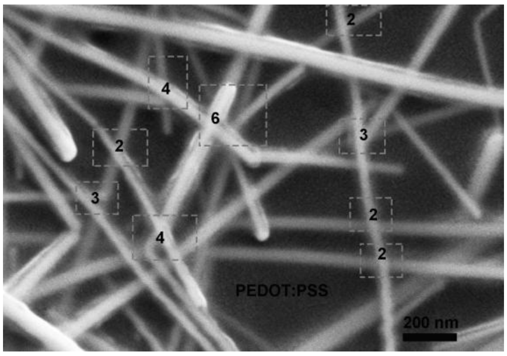

Figure S9. SEM images of Ag NWs in the PEDOT:PSS/PVA/Ag NWs film.

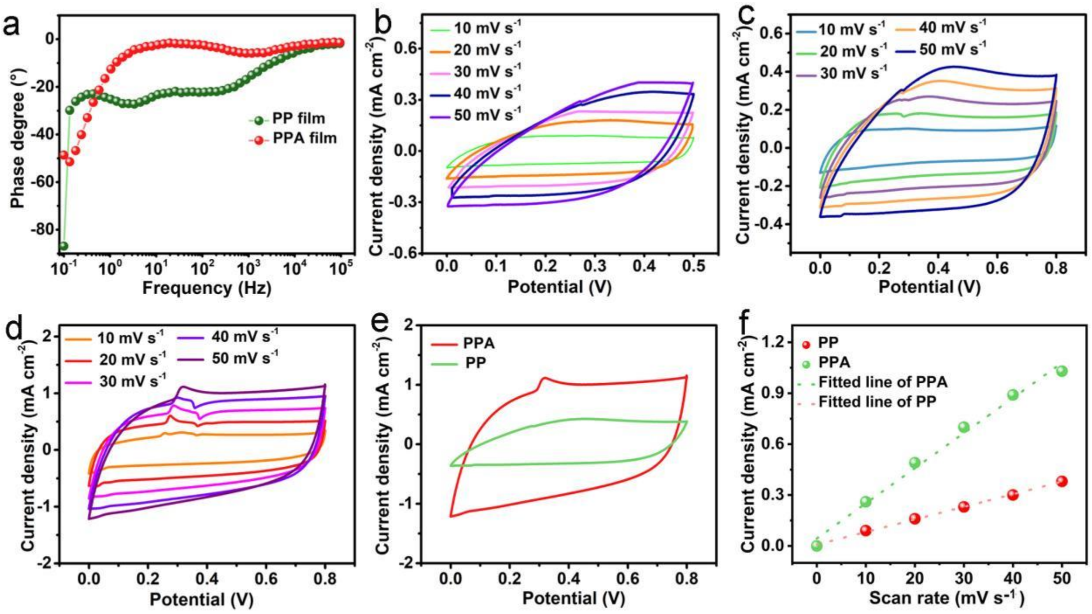

Figure S10. (a) Phase degree-frequency diagram of PEDOT:PSS/PVA and PEDOT:PSS/PVA/Ag NWs film. (b) CVs of PEDOT:PSS/PVA films as electrodes at different scan rates (0-0.5V). (c, d) CVs of PEDOT:PSS/PVA (c) and PEDOT:PSS/PVA/Ag NWs films (d) at different scan rates in 0.01 M PBS (0-0.8V). (e) CVs of PEDOT:PSS/PVA and PEDOT:PSS/PVA/Ag NWs films as electrodes at a scan rate of 50 mV s^{-1}. (f) Linear relationship between charging current and scan rate for PEDOT:PSS/PVA films and PEDOT:PSS/PVA/Ag NWs films in 0.01 M PBS (0-0.8V).

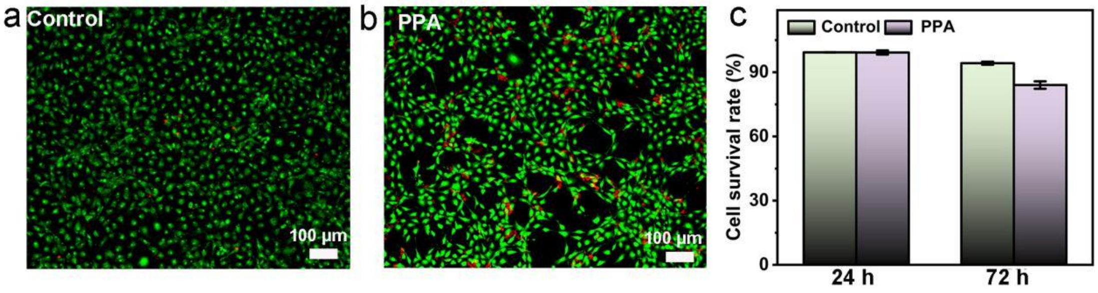

Figure S11. (a, b) Fluorescent images of stained NIH 3T3 cells cultured on control substrate and PEDOT:PSS/PVA/Ag NWs film after 72h. Live cells: green. Dead cells: red. (c) Cell viability rate at 24 h and 72 h of incubation.

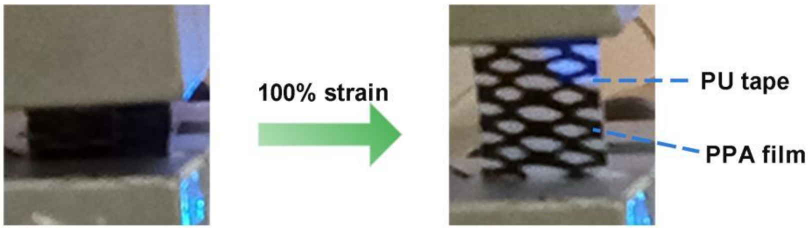

Figure S12. Digital images of Kirigami-structured PEDOT:PSS/PVA/Ag NWs/PU electrodes during stretching.

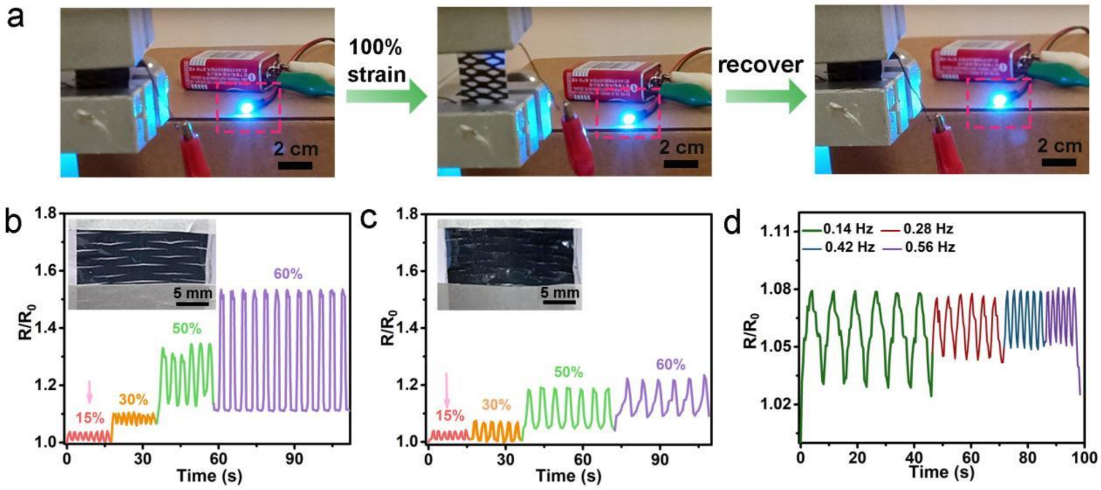

Figure S13. (a) Digital images of brightness changes of LED light during the stretch-release process of the Kirigami-structured PEDOT:PSS/PVA/Ag NWs/PU electrode. (b, c) Resistance changes of the Kirigami-structured PEDOT:PSS/PVA/Ag NWs/PU electrodes fabricated without prestretch process (b) and with prestretch process (c) at various strains, respectively. Insets are the digital images of the corresponding electrodes. (d) Resistance change of the Kirigami-structured PEDOT:PSS/PVA/Ag NWs/PU electrode under 30% strain at diverse frequencies.

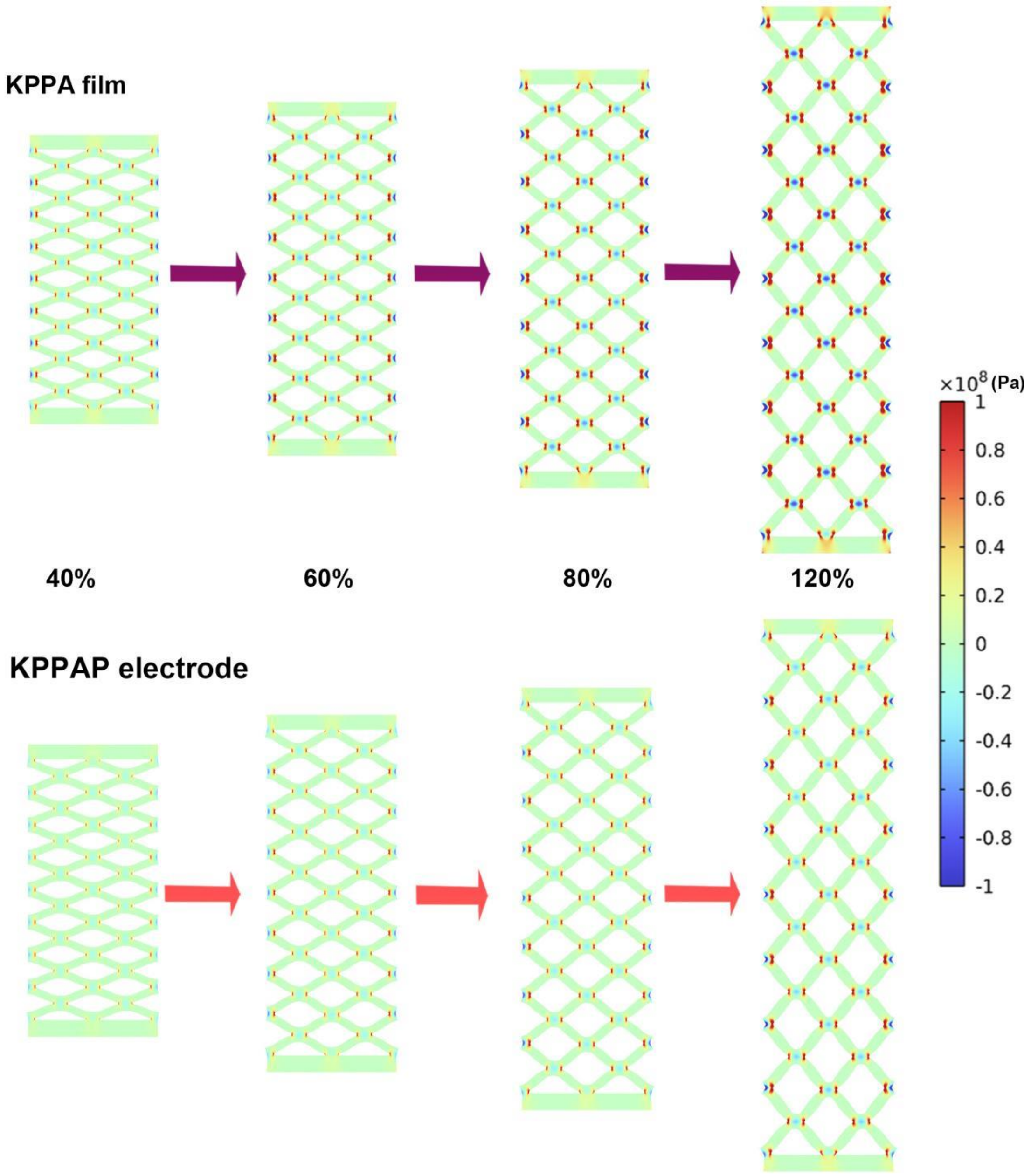

Figure S14. Simulated stress distributions of the Kirigami-structured PEDOT:PSS/PVA/Ag NWs film and Kirigami-structured PEDOT:PSS/PVA/Ag NWs/PU electrode at different strains. When same strains are exerted, Kirigami-structured PEDOT:PSS/PVA/Ag NWs/PU electrodes bear less stress at the fracture points due to the protection of PU tape, and it is more difficult to break, preventing damage to the conductive network.

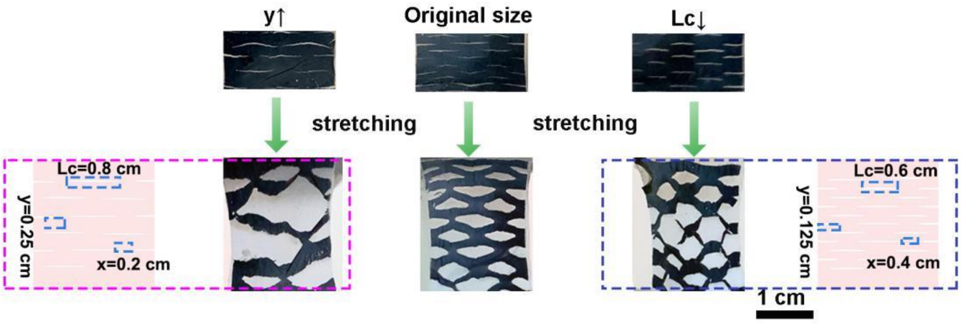

Figure S15. Stretching processes of Kirigami patterns with different cutting sizes.

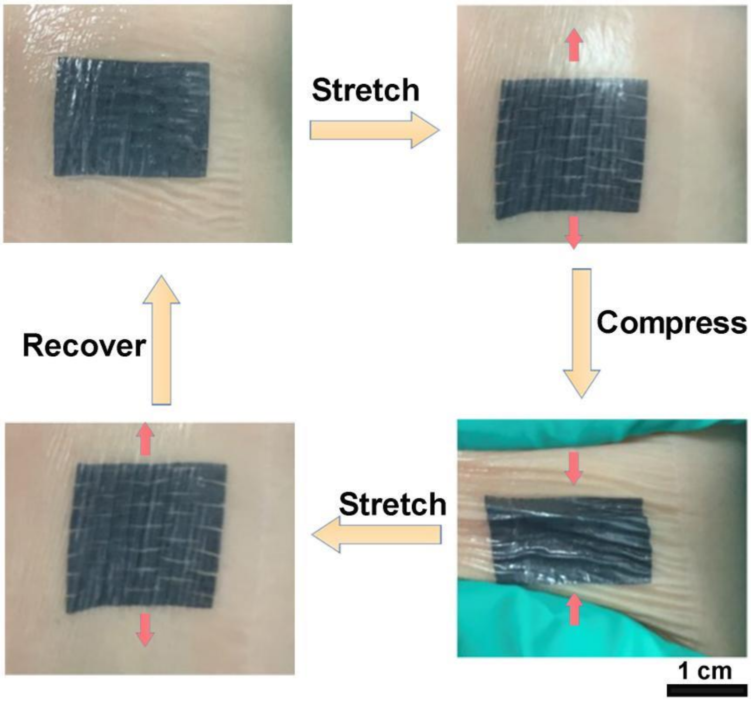

Figure S16. Top view of a Kirigami-structured PEDOT:PSS/PVA/Ag NWs/PU electrode on skin with different deformation.

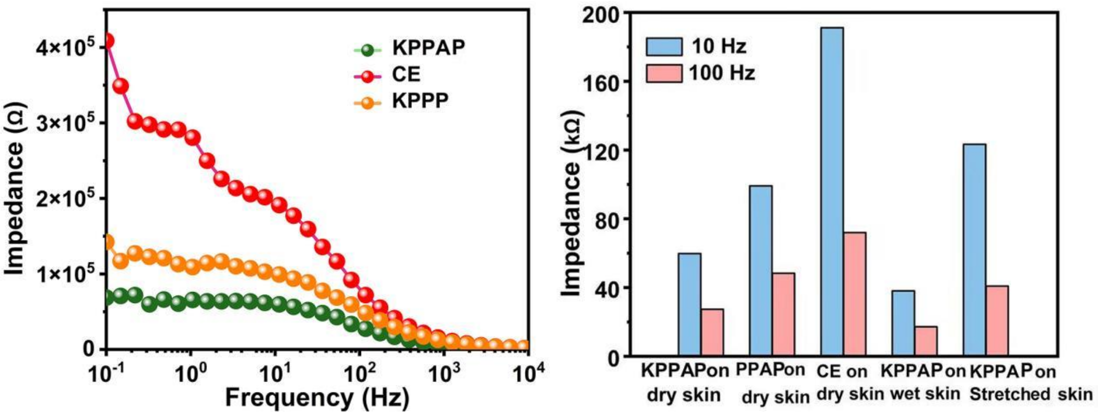

Figure S17. (a) Interfacial impedance of different electrode-skin systems from 0.1 to 10^5 Hz. (b) Comparison of interfacial impedance of different electrode-skin systems in different conditions at 10 Hz and 100 Hz.

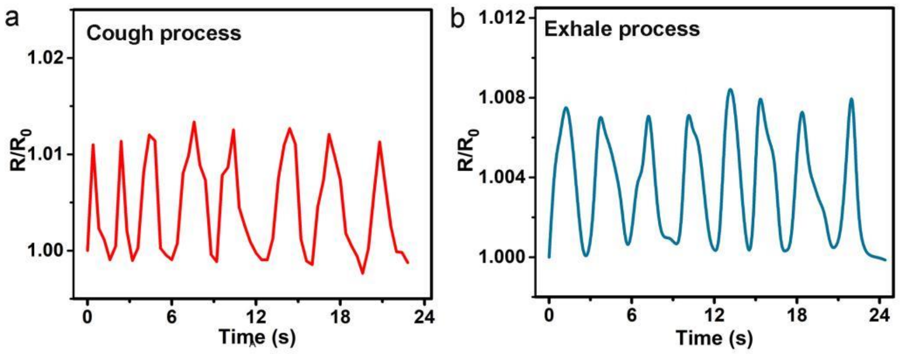

Figure S18. Kirigami-structured PEDOT:PSS/PVA/Ag NWs/PU electrode-based strain sensors for (a) detecting cough process, (b) exhale process.

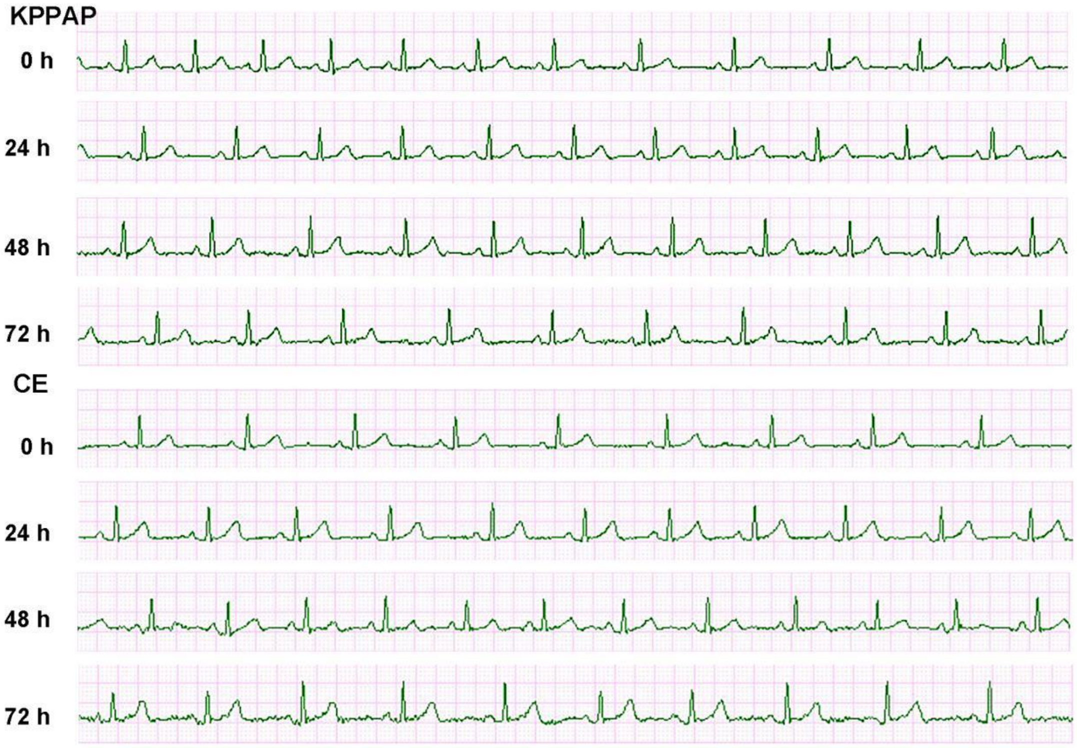

Figure S19. Long-term ECG signal recording at different time using Kirigami-structured PEDOT:PSS/PVA/Ag NWs/PU electrodes and commercial electrodes at 0 h, 24 h, 48 h and 72 h, respectively.

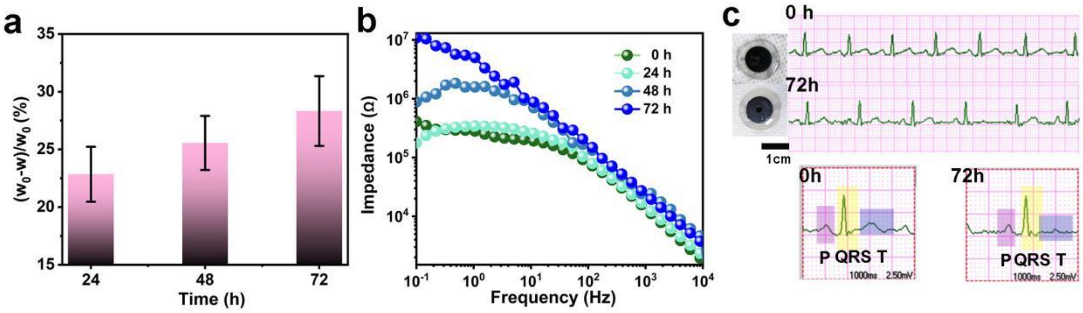

Figure S20. (a) Weight loss percentage of commercial electrodes placed at room temperature for different time. (b) Interfacial impedance of commercial electrodes placed at room temperature for different times. (c) ECG signals recorded with commercial electrodes placed for 72 hours and 0 hours.

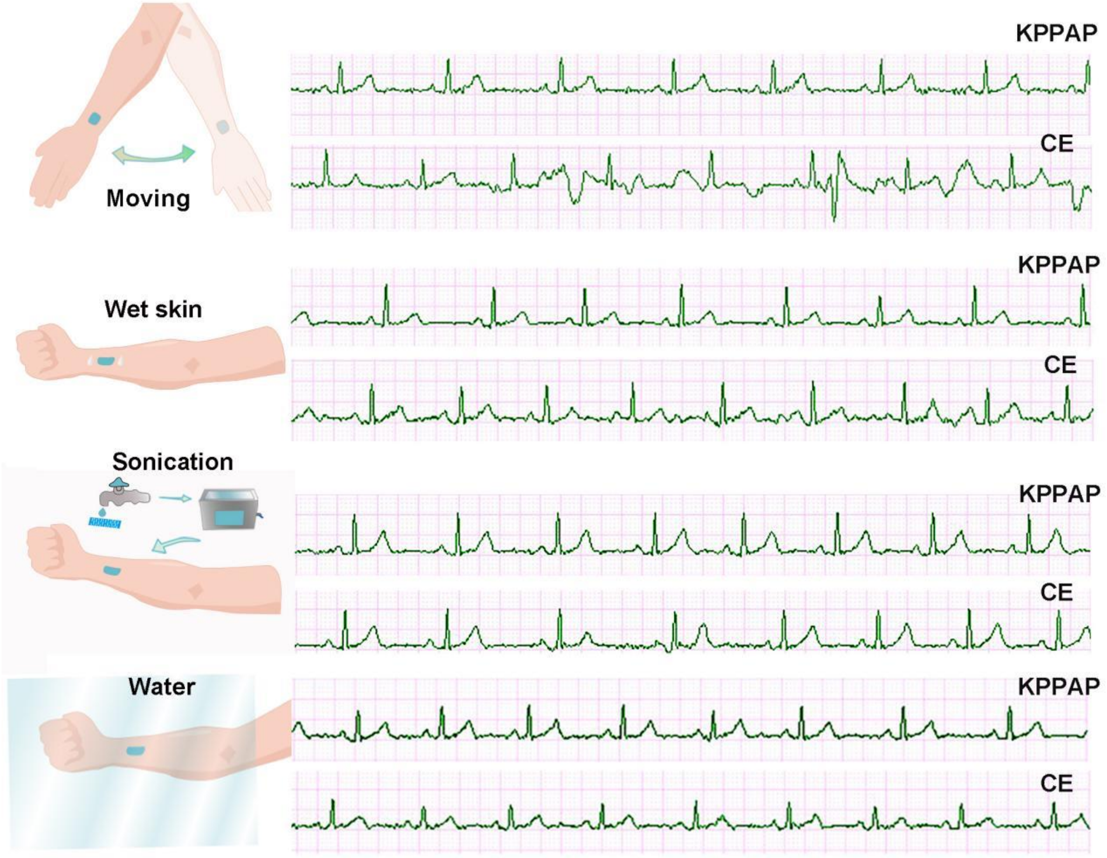

Figure S21. ECG signals recorded using Kirigami-structured PEDOT:PSS/PVA/Ag NWs/PU electrodes and commercial electrodes under different circumstances (e.g., moving, wet skin, sonication, and under water).

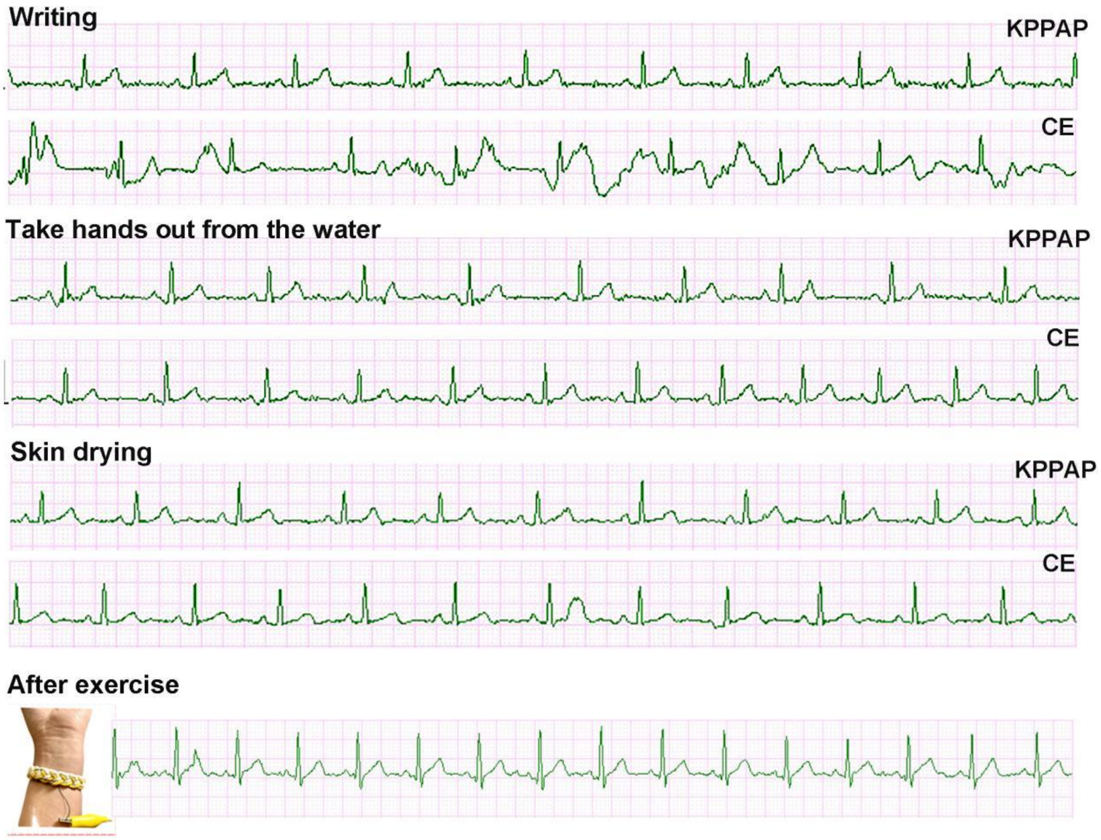

Figure S22. ECG signals recorded by Kirigami-structured PEDOT:PSS/PVA/Ag NWs/PU electrodes and commercial electrodes after arms are immersed in water, removed from water, and dried, and ECG signals recorded by wristband after exercise.

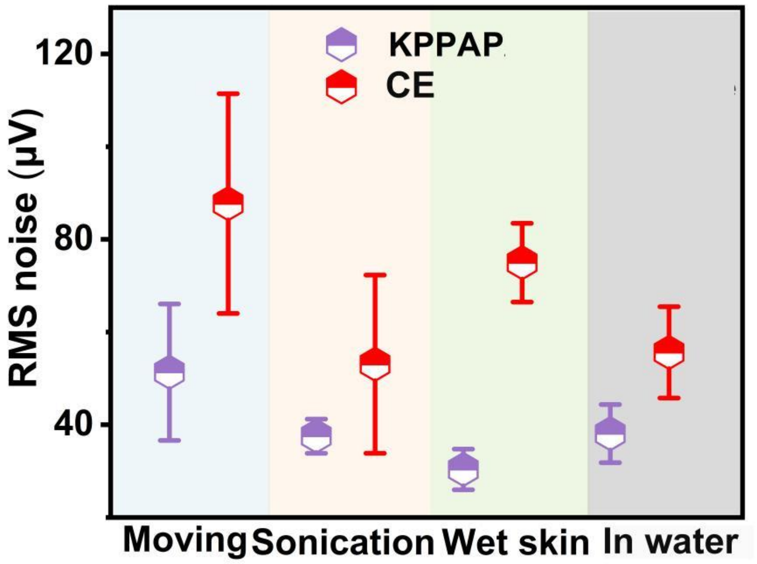

Figure S23. The RMS noise calculated from the baseline between T and P waves of ECG signals picked by Kirigami-structured PEDOT:PSS/PVA/Ag NWs/PU electrodes and commercial electrodes during ECG recording under different circumstances (e.g., moving, wet skin, sonication, and under water).

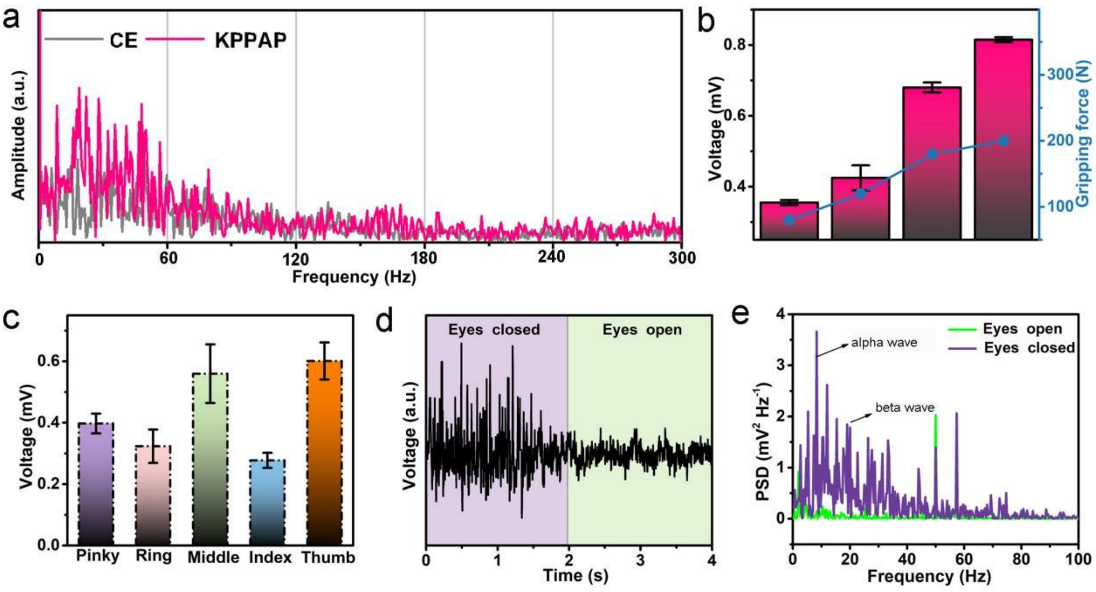

Figure S24. (a) Spectra of the EMG pulse recorded using the Kirigami-structured PEDOT:PSS/PVA/Ag NWs/PU electrode and commercial electrodes. (b) Variations of

the EMG signal amplitude with different gripping force. (c) EMG signal amplitude produced by the flexion/extension of different fingers. (d) Time-domain EEG signals of open eyes and closed eyes recorded by the Kirigami-structured PEDOT:PSS/PVA/Ag NWs/PU electrode. (e) Spectra of the EEG signal with eyes open and eyes closed.

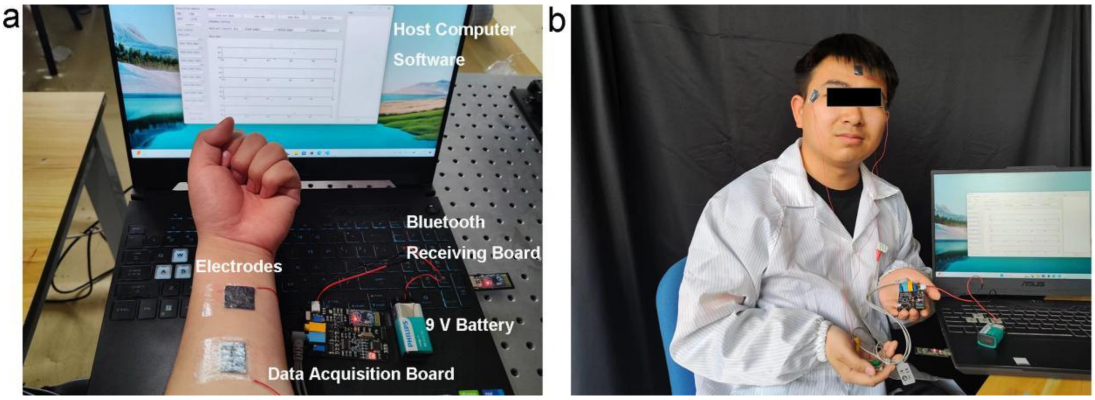

Figure S25. (a, b) Layout of circuit with the Kirigami-structured PEDOT:PSS/PVA/Ag NWs/PU electrodes for applications in the operations of music play/switch using EOG signals (a) and snake game using EMG signals (b).

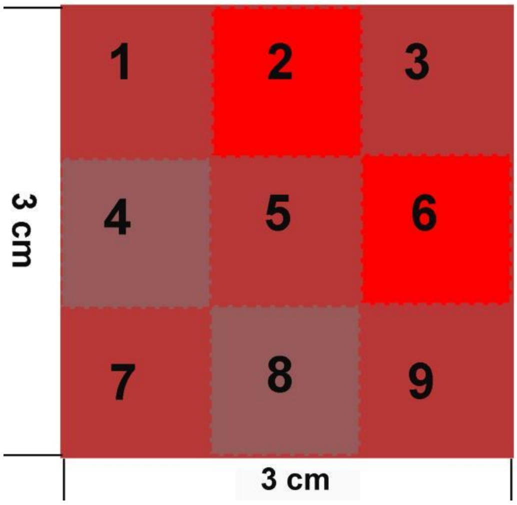

Figure S26. Measured points of sheet resistance on one sample.

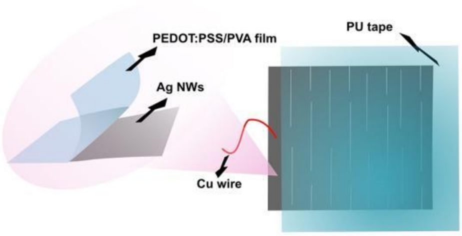

Figure S27. Method of metal wires connected to epidermal electrodes.

Table S1. Comparison of our kirigami-structured PEDOT:PSS/PVA/Ag NWs/PU electrodes with the reported electrodes

Electrodes (major constituents, thickness)	Interfacial Impedance	Conductivity	Sensitivity	Stretch-ability	Ref
PPT (PEDOT:PSS, PVA , TA; 20–25 μm)	256 kΩ cm^2 (10 Hz, *)	122 S cm^{-1}	0.15 (30 %)	54%	1
Porous HP-Ag NW/TPU (Ag NWs, TPU; 4.6 μm)	200 kΩ (10 Hz, *)	7.3 Ω sq^{-1}	7 (15 %)	45%	2
PWS (PEDOT:PSS, WPU, D-sorbitol; 20 μm)	82 kΩ cm^2 (10 Hz, 7.065 cm^2)	545 S cm^{-1}	0.055 (<30 %)	43%	3
PAP (PEDOT:PSS, Ag NWs; 230 nm)	>150 kΩ (10 Hz, 1 cm^2)	16.8 Ω sq^{-1}	*	*	4
Silk-P-15G (silk fibroin films, PEDOT:PSS, glycerol, Triton X-100; 8~10 μm)	~400 kΩ (10 Hz, 3.14 cm^2)	24 S cm^{-1}	<0.2 (30 %)	250%	5
Janus textile electrode (PU, Ag NWs; MA, ~15 μm)	~300 kΩ (10 Hz, 2 cm^2)	4.3 Ω sq^{-1}	*	> 500%	6
CNFF-based kirigami (ANF, PVA, PEDOT, CPD; ~100 μm)	120–180 kΩ (100 Hz, *)	*	Nearly 0 (130 %)	~180%	7
PDMS-Ag NW(Triton X, PDMS, Ag NWs; 1 mm)	~100 kΩ (10 Hz, 6.25 cm^2)	35 Ω sq^{-1}	1.6 (20 %)	>400%	8

Au/TPU/CM (cellulose membrane, cellulose acetate, TPU, Au; a few micrometers)	>200 kΩ (10 Hz, 4 cm^2)	2.68 Ω sq^{-1}	2 (19%)	*	[9]
MPM (Mxene, PU; <20 μm)	>80 kΩ (10 Hz, *)	*	~4 (30%)	>40%	[10]
SESA-based epidermal Patches (ALA, MBAA, $FeCl_3$, CNT; 140 μm)	>200 kΩ (10 Hz, *)	134 Ω sq^{-1}	1.3 (50%)	>600%	[11]
KPPA (PEDOT:PSS, Ag NWs, PVA; 4.1 μm)	59 kΩ (10 Hz, 3 cm^2)	3.9 Ω sq^{-1}	0.082 (30 %)	>100%	This work

(* means did not mentioned in the article)

Table S2. Sample Naming Explanation

Abbreviated names	Full names	With or without prestretch process	With or without kirigami structured
PP film	PEDOT: PSS /PVA	Without	No
PEDOT:PSS/PVA/Ag NWs film	PEDOT: PSS/PVA/Ag NWs	Without	No
Kirigami-structured PEDOT:PSS/PVA electrode	PEDOT: PSS/PVA/PU tape	With	Yes
Kirigami-structured PEDOT:PSS/PVA/Ag NWs/PU electrode	PEDOT: PSS/PVA/Ag NWs/PU tape	With	Yes

REFERENCES

[1] J. Cao, X. Yang, J. Rao, A. Mitriashkin, X. Fan, R. Chen, H. Cheng, X. Wang, J. Goh, H.L. Leo, J. Ouyang, *ACS Appl. Mater. Interfaces* **2022**, *14*, 39159.

[2] W. Zhou, S. Yao, H. Wang, Q. Du, Y. Ma, Y. Zhu, *ACS Nano* **2020**, *14*, 5798.

[3] L. Zhang, K.S. Kumar, H. He, C.J. Cai, X. He, H. Gao, S. Yue, C. Li, R.C. Seet, H. Ren, J. Ouyang, *Nat. Commun.* **2020**, *11*, 4683.

[4] Y. Fang, Y. Li, Y. Li, M. Ding, J. Xie, B. Hu, *ACS Appl. Mater. Interfaces* **2020**, *12*, 23689.

[5] Q. Li, G. Chen, Y. Cui, S. Ji, Z. Liu, C. Wan, Y. Liu, Y. Lu, C. Wang, N. Zhang, Y. Cheng, K.Q. Zhang, X. Chen, *ACS Nano* **2021**, *15*, 9955.

[6] X. Yang, S. Wang, M. Liu, L. Li, Y. Zhao, Y. Wang, Y. Bai, Q. Lu, Z. Xiong, S. Feng, T. Zhang, *Small* **2022**, *18*, e2106477.

[7] H. Liu, H. Li, Z. Wang, X. Wei, H. Zhu, M. Sun, Y. Lin, L. Xu, *Adv. Mater.* **2022**, *34*, 2207350.

[8] J.H. Kim, S.R. Kim, H.J. Kil, Y.C. Kim, J.W. Park, *Nano. Lett.* **2018**, *18*, 4531.

[9] Y. Xu, W. Guo, S. Zhou, H. Yi, G. Yang, S. Mei, K. Zhu, H. Wu, Z. Li, *Adv. Funct. Mater.* **2022**, *32*, 2200961.

[10] T. Cui, Y. Qiao, D. Li, X. Huang, L. Yang, A. Yan, Z. Chen, J. Xu, X. Tan, J. Jian, Z. Li, S. Ji, H. Liu, Y. Yang, X. Zhang, T.-L. Ren, *Chem. Eng. J.* **2023**, *455*, 140690.

[11] Y. Cheng, Y. Zhou, R. Wang, K.H. Chan, Y. Liu, T. Ding, X.Q. Wang, T. Li, G.W. Ho, *ACS Nano* **2022**, *16*, 18608.
